# Morphology modulation of artificial muscles by thermodynamic-twist coupling

**DOI:** 10.1093/nsr/nwac196

**Published:** 2022-09-22

**Authors:** Xiaoyu Hu, Jiatian Li, Sitong Li, Guanghao Zhang, Run Wang, Zhongsheng Liu, Mengmeng Chen, Wenqian He, Kaiqing Yu, Wenzhong Zhai, Weiqiang Zhao, Abdul Qadeer Khan, Shaoli Fang, Ray H Baughman, Xiang Zhou, Zunfeng Liu

**Affiliations:** State Key Laboratory of Medicinal Chemical Biology, College of Chemistry and College of Pharmacy, Key Laboratory of Functional Polymer Materials, Frontiers Science Center for New Organic Matter, Nankai University, Tianjin 300071, China; State Key Laboratory of Medicinal Chemical Biology, College of Chemistry and College of Pharmacy, Key Laboratory of Functional Polymer Materials, Frontiers Science Center for New Organic Matter, Nankai University, Tianjin 300071, China; State Key Laboratory of Medicinal Chemical Biology, College of Chemistry and College of Pharmacy, Key Laboratory of Functional Polymer Materials, Frontiers Science Center for New Organic Matter, Nankai University, Tianjin 300071, China; State Key Laboratory of Medicinal Chemical Biology, College of Chemistry and College of Pharmacy, Key Laboratory of Functional Polymer Materials, Frontiers Science Center for New Organic Matter, Nankai University, Tianjin 300071, China; State Key Laboratory of Medicinal Chemical Biology, College of Chemistry and College of Pharmacy, Key Laboratory of Functional Polymer Materials, Frontiers Science Center for New Organic Matter, Nankai University, Tianjin 300071, China; State Key Laboratory of Medicinal Chemical Biology, College of Chemistry and College of Pharmacy, Key Laboratory of Functional Polymer Materials, Frontiers Science Center for New Organic Matter, Nankai University, Tianjin 300071, China; State Key Laboratory of Medicinal Chemical Biology, College of Chemistry and College of Pharmacy, Key Laboratory of Functional Polymer Materials, Frontiers Science Center for New Organic Matter, Nankai University, Tianjin 300071, China; State Key Laboratory of Medicinal Chemical Biology, College of Chemistry and College of Pharmacy, Key Laboratory of Functional Polymer Materials, Frontiers Science Center for New Organic Matter, Nankai University, Tianjin 300071, China; State Key Laboratory of Medicinal Chemical Biology, College of Chemistry and College of Pharmacy, Key Laboratory of Functional Polymer Materials, Frontiers Science Center for New Organic Matter, Nankai University, Tianjin 300071, China; State Key Laboratory of Medicinal Chemical Biology, College of Chemistry and College of Pharmacy, Key Laboratory of Functional Polymer Materials, Frontiers Science Center for New Organic Matter, Nankai University, Tianjin 300071, China; State Key Laboratory of Medicinal Chemical Biology, College of Chemistry and College of Pharmacy, Key Laboratory of Functional Polymer Materials, Frontiers Science Center for New Organic Matter, Nankai University, Tianjin 300071, China; State Key Laboratory of Medicinal Chemical Biology, College of Chemistry and College of Pharmacy, Key Laboratory of Functional Polymer Materials, Frontiers Science Center for New Organic Matter, Nankai University, Tianjin 300071, China; Alan G. MacDiarmid NanoTech Institute, University of Texas at Dallas, Richardson, TX 75080, USA; Alan G. MacDiarmid NanoTech Institute, University of Texas at Dallas, Richardson, TX 75080, USA; Department of Science, China Pharmaceutical University, Nanjing 211198, China; State Key Laboratory of Medicinal Chemical Biology, College of Chemistry and College of Pharmacy, Key Laboratory of Functional Polymer Materials, Frontiers Science Center for New Organic Matter, Nankai University, Tianjin 300071, China

**Keywords:** artificial muscle, actuator, twist, functional fibres, bio-mimetic, thermodynamic-twist coupling, smart materials

## Abstract

Human muscles can grow and change their length with body development; therefore, artificial muscles that modulate their morphology according to changing needs are needed. In this paper, we report a strategy to transform an artificial muscle into a new muscle with a different morphology by thermodynamic-twist coupling, and illustrate its structural evolution during actuation. The muscle length can be continuously modulated over a large temperature range, and actuation occurs by continuously changing the temperature. This strategy is applicable to different actuation modes, including tensile elongation, tensile contraction and torsional rotation. This is realized by twist insertion into a fibre to produce torsional stress. Fibre annealing causes partial thermodynamic relaxation of the spiral molecular chains, which serves as internal tethering and inhibits fibre twist release, thus producing a self-supporting artificial muscle that actuates under heating. At a sufficiently high temperature, further relaxation of the spiral molecular chains occurs, resulting in a new muscle with a different length. A structural study provides an understanding of the thermodynamic-twist coupling. This work provides a new design strategy for intelligent materials.

## Teaser

The artificial muscle with precise reversibility control by thermodynamic-twist coupling is applicable to different actuation modes, including elongation, contraction, and torsional rotation.

## INTRODUCTION

Artificial muscles that contract, elongate or rotate when externally stimulated [1–7] have emerging applications in soft robotics [8–11], smart textiles [12,13], intelligent control [14], sensing [15], etc. [16–19]. Human muscles grow in length during body development, and development of artificial muscles that can similarly modulate their morphology and actuation, but on a much shorter time scale, is desirable. Such modification of artificial muscle structure and properties would eliminate the need to replace muscles when environmental needs change. It is still a challenge for an artificial muscle to precisely modulate its length.

Twisted fibres and yarns have been used as torsional and tensile artificial muscles, whose actuation is driven by amplification of the radial expansion and axial contraction by the spiral architecture of the muscle fibre or yarn [20–22]. Twisted yarn and fibre muscles provide highly reversible torsional and tensile actuation by torsional tethering [23] or irreversible actuation without torsional tethering [24]. During twist insertion, the molecular chains get aligned and torsional stress is generated, and during volume expansion the fibre tends to release the inserted twist, resulting in torsional and tensile actuation. Therefore, the twist as well as the generated torsional stress are key factors in the actuation performances of artificial muscles. Up to now, it has been difficult to achieve characterization and modulation of the torsional molecular alignment and torsional stress in the twisted polymer fibre artificial muscles.

**Figure 1.**
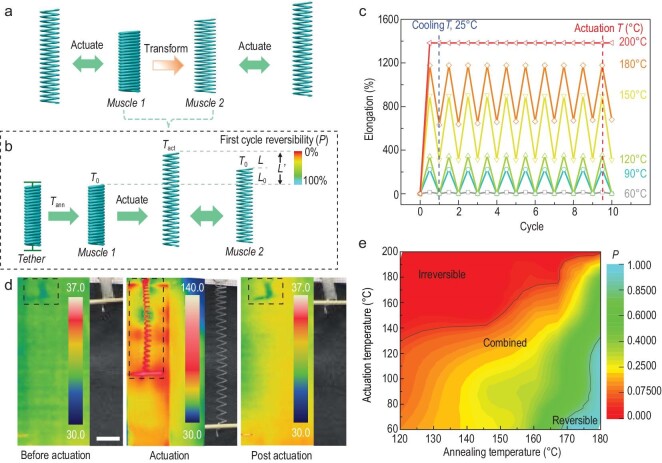
Coiled heterochiral nylon 6 fibre artificial muscles with multimodal tensile actuation. (a and b) Schematic illustration of morphological modulation by thermodynamic-twist coupling, employing a heterochiral fibre muscle. (c) Elongation as a function of the number of heating/cooling cycles for the artificial muscles with irreversible, reversible and combined irreversible and reversible actuation, for an annealing temperature of 180°C. (d) Optical and thermal images of the 0.4-mm-diameter heterochiral coiled nylon 6 fibre muscle with reversible actuation mode before, during and after actuation, for a highest infrared-measured actuation temperature of ∼120°C. A lightweight (<0.03 g) paddle was attached to prevent rotation during annealing. Scale bar: 1 cm. (e) Reversibility factor (*P*) as a function of thermal annealing and actuation temperatures for the artificial muscles.

In this work, we monitored the evolution of molecular chain alignment and the torsional stress of artificial muscle during actuation via 2D X-ray diffraction (XRD). Then we developed a thermodynamic-twist coupling strategy to realize artificial muscle fibres that can modulate their length. This was realized by annealing a twisted artificial muscle fibre at different temperatures to allow a certain part of the polymer chain segments to relax their torsional stress, which serves as an internal return spring to balance the rest of the molecular chain segments. The resulting artificial muscles exhibit different reversibility depending on the different actuation temperatures. The reversibility control lies in the difference in the retention of the internal torsional stress, which inspired us to design an artificial muscle that can transform into a new muscle with a different length by regulating the reversibility via a thermodynamic-twist coupling strategy (Fig. 1a)and b).

Briefly, the spiral molecular chains were modulated by the thermodynamic relaxation in the twisted, coiled fibres (with coil length *L*_0_). Twist insertion into a polymer fibre generates internal stress, and thermal annealing (at *T*_ann_) produces a self-supporting artificial muscle, where heating-induced partial thermodynamic relaxation of molecular chains can inhibit fibre twist release [25,26]. This self-supporting artificial muscle can actuate under heating (*T*_act_), while for a sufficiently high *T*_act_, further relaxation of the molecular chains occurs, transforming the muscle into another muscle with a different return coil length (*L’*) (Fig. 1b). The relative values of *T*_ann_ and *T*_act_ affect the actuation reversibility, i.e. increasing *T*_ann_ or decreasing *T*_act_ increases the reversibility, and vice versa. Consequently, the length of this new muscle can be continuously modulated by tuning *T*_ann_ and *T*_act_. This strategy is applicable to many semicrystalline polymer fibres, such as nylon 6, nylon 6, 6, and polyethylene (PE), for different morphological modulations, such as elongation, contraction and rotation. Reversibility control and morphology modulation were demonstrated in sensors, intelligent controllers and soft robots [27].

## RESULTS AND DISCUSSION

### Multimodal artificial muscles from twisted, coiled nylon 6 fibres

We first demonstrated multimodal artificial muscles by using a nylon 6 monofilament fishing line fibre, which was employed for morphological modulation (Fig. 1a). This 0.45-mm-diameter nylon 6 fibre was twisted with a twist density of 5.4 turns cm^−1^ in the S direction (counterclockwise) and wrapped around a 3-mm-diameter steel rod in the Z direction (clockwise) to form a heterochiral coil. The coil was both-end tethered and annealed at 180°C for 1 h. Unless otherwise specified, this annealing time was used for other similar annealing processes. The shape of the coil was retained after cooling to room temperature (25°C) and removing the tethering (Fig. 1b). Heating this coil to 60°C, without load or torsional tethering, resulted in coil elongation by 14% (tensile actuation strain), and the coil returned to the initial length upon cooling to room temperature (Fig. 1c). Unless otherwise specified, all the described muscles were not torsionally tethered during actuation, which is important for obtaining multimodal actuation and morphology modulation. The tensile actuation strain (ϵ) is ϵ = (*L*−*L*_0_)/*L*_0_, where *L*_0_ and *L* are the muscle lengths before and during actuation, respectively. Since the above fibre is heterochiral, transfer of the thermal-expansion-produced down-twist of the fibre to the twist of the coil results in a down-twist in the twist of the coil and corresponding muscle elongation [23]. This reversible process could be repeated 7000 times (Fig. S1).

Increasing this heating temperature (also called the actuation temperature) to 120°C increased the coil elongation to 347%, and the muscle still showed fully reversible actuation. Heating this coil to 180°C led to coil elongation by 1180% (corresponding to 12.8*L*_0_), and the coil returned to 730% of the initial coil length (corresponding to 7.3*L*_0_) upon cooling to room temperature; this coil elongated and contracted between these two lengths (12.8*L*_0_ and 7.3*L*_0_) in subsequent heating–cooling cycles. This is partially reversible actuation, and in this case, the muscle length is modulated since the new muscle length (7.3*L*_0_) is different from the initial muscle length (*L*_0_). Heating this heterochiral coil to 200°C further increased the elongation to 1380%, and the coil remained at this elongated length upon subsequent cooling to room temperature, which was irreversible actuation.

Such multimodal actuation was observed over a wide range of thermal annealing temperatures (120–180°C) and actuation temperatures (60–200°C) for this nylon 6 fibre (Fig. 1e)and Fig. S2). To quantitatively describe the reversible actuation degree of the twist-based artificial muscles, we defined the reversible stroke (ϵ_r_) as the length change ratio before and after reversible actuation, ϵ_r_ = (*L*−*L*′)/*L*′, where *L*′ and *L* are the lengths before and after reversible actuation, respectively. Similarly, we defined the irreversible stroke (ϵ_ir_) as the length change ratio before and after irreversible actuation, ϵ_ir_ = (*L*′−*L*_0_)/*L*_0_. Note that irreversible and reversible actuation coexist if *L*′ does not equal *L*_0_. Therefore, we defined a reversibility factor (*P*) to describe the fraction of the total actuation stroke that is reversible, *P* = ϵ_r_/(ϵ_r_ + ϵ_ir_), as shown in Fig. S3b. Figure 1e)shows that this reversibility factor increases with increasing thermal annealing temperature *T*_ann_ and decreases with increasing actuation temperature *T*_act_, as calculated from the data in Fig. S4.

### Structural evolution during thermodynamic-twist coupling

To understand the mechanism of reversibility modulation of artificial muscles by thermodynamic-twist coupling, we next studied the structural evolution of the crystalline and amorphous regions in the twisted fibres during thermal annealing and thermoactuation. Unless otherwise specified, thermal annealing was carried out at different temperatures for 1 h, and all the structural characterizations were carried out at room temperature after thermal annealing or actuation (Supplementary Note 4).

We first investigated the shape fixation of the twist-containing fibres by thermal annealing (Supplementary Note 4.1). A twisted nylon 6 fibre heterochirally coiled around a mandrel would extend to release the internal torsional stress upon release of the tethering, and thermal annealing of a tethered coil decreased the extension after the release of the tethering. Here, the shape fixity is employed to evaluate the extent of shape fixation by thermal annealing, which is defined as the ratio of the tethered coil length obtained by mandrel coiling to the non-tethered coil length after annealing (and after 24 h at room temperature) (Fig. S12a). The shape fixity increased with annealing temperature and reached 99% for an annealing temperature of 180°C. Similarly, a twisted fibre would show decreased length (*L*_T_) and increased diameter (*R*_T_) with twist insertion (Supplementary Note 4.2). After annealing a twisted fibre at 180°C, negligible changes were observed for the *L*_T_/*L*_0_ and *R*_T_/*R*_0_ ratios with time, indicating that negligible fibre twist release occurred (Fig. S13).

**Figure 2.**
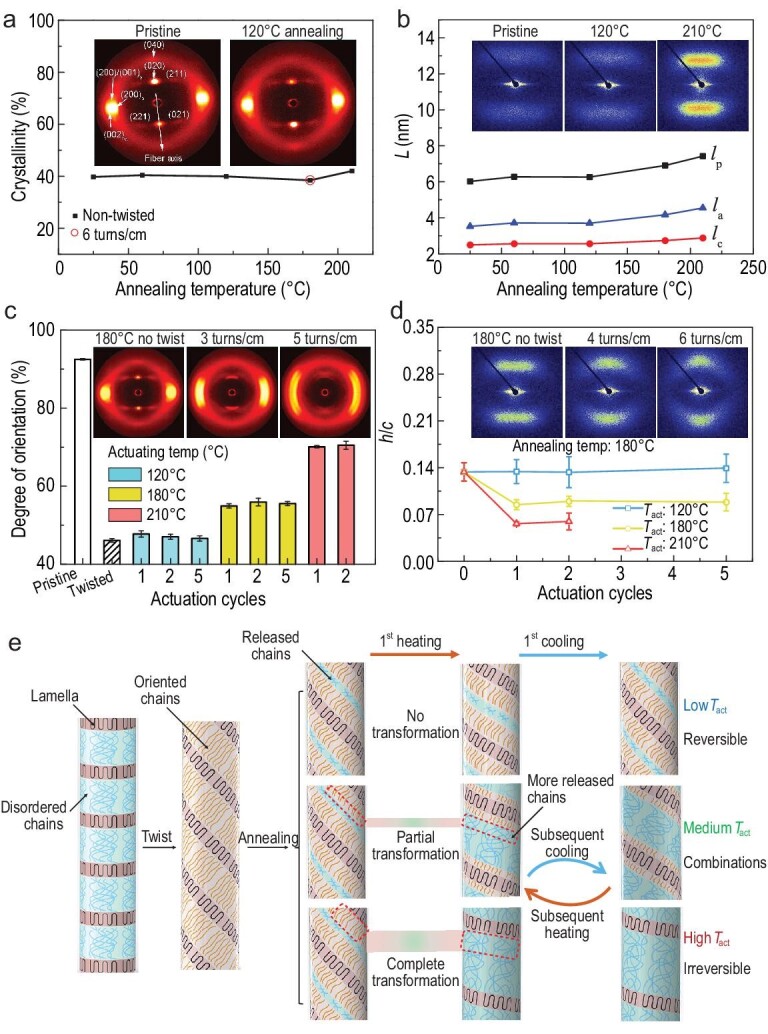
Microstructural evolution for nylon 6 fibre muscles by thermodynamic-twist coupling. (a) Crystallinity for tethered nylon 6 fibres that have different annealing temperatures as measured by DSC. Inset: the 2D WAXS patterns. (b) The *l*_a_, *l*_p_ and *l*_c_ for the non-twisted fibres at different annealing temperatures. Inset: 2D SAXS patterns for non-twisted fibres that have different annealing temperatures. (c) DOC of the fibre muscles before actuation and after actuation at 120 and 210°C for different numbers of cycles, using a pristine fibre as a control. The inserted twist was 14.0 turns cm^−1^. Inset: 2D WAXS patterns for the fibre muscles with different twist densities. (d) The *h*/*c* for twisted fibre muscles, before actuation and after actuation at 120, 180 and 210°C for different numbers of cycles. The twist density was 6.0 turns cm^−1^. Inset: 2D SAXS scattering patterns for the fibre muscles with different twist densities. (e) Schematic illustration of the microstructural evolution for the artificial muscles with reversibility modulation. In (c) and (d), the error bars depict the standard deviation for a sample number of three, and the annealing temperature is 180°C. For (a) to (d), the fibre diameter is 0.4 mm unless otherwise indicated. All XRD measurements were conducted at room temperature.

We next investigated the thermal setting of the spirally oriented crystalline and amorphous regions of the twisted fibres by employing wide-angle X-ray scattering (WAXS) measurements (Supplementary Note 4.3). A pristine nylon 6 fibre exhibited strong arc-shaped diffraction patterns in the axial direction in WAXS measurements (Fig. 2a, inset), indicating alignment of the molecular chains in the crystallites along the axial direction. Inserting a twist increased the arc length, indicating that the spiral architecture of the molecular chains decreased their axial alignment (Fig. S14, inset) [20–22]. Herein, the degree of orientation of crystals (DOC), calculated as (180°−θ_h_)/180°, was used to evaluate the alignment of molecular chains in the fibre, where θ_h_ is the arc angle at the half-maximum of the integrated diffraction intensity from WAXS patterns (Fig. S15). The DOC increased with time as a non-annealed twisted fibre was left untethered at room temperature (Fig. S16a), indicating untwisting of the crystals and stress relaxation in this fibre. Thermal annealing of the tethered twisted nylon 6 fibre decreased this crystal untwisting at room temperature, and negligible untwisting was observed for the twisted fibre with an annealing temperature of 180°C (Fig. S16b).

Such an alignment change of the crystal region of a twisted fibre during thermal setting can also be revealed by small-angle X-ray scattering (SAXS, Supplementary Note 4.4). The pristine nylon 6 fibre exhibited two quasi-rectangular scattering spots in the axial direction (Fig. 2b, inset), which became an arc shape for a twisted fibre. Herein, the alignment of the crystals can be expressed as the ratio of the sagitta (*h*) of the inner arc to the corresponding chord length (*c*), *h*/*c*, where a larger twist density resulted in a higher *h*/*c* and consequently a decreased crystal alignment (Fig. S17). Thermal annealing of the twisted fibre at 180°C resulted in a negligible change in *h*/*c* with time, indicating preservation of the spirally aligned crystals in the fibre.

The twist evolution of the artificial muscles with different reversibility during thermal actuation was characterized by employing DOC (WAXS) and *h*/*c* (SAXS) (Fig. 2c)and d, Supplementary Notes 4.5 and 4.6). Negligible changes in DOC and *h*/*c* were observed after five cycles of reversible actuation at 120°C for a twisted nylon 6 artificial muscle with an annealing temperature of 180°C, indicating no twist loss in the molecular chains and crystallites. After multimodal actuation at 180°C and irreversible actuation at 210°C, *h*/*c* decreased from 0.134 to 0.085 and 0.056, and the DOC increased from 46.1% to 54.9% and 70.1%, respectively, indicating that twist loss of molecular chains resulted in decreased reversibility. Similar structural changes were also observed for the artificial muscles with an annealing temperature of 150°C and different actuation temperatures (Fig. S18). The above results indicate that the reversibility modulation is highly correlated with the twist modulation of the stressed molecular chains during thermodynamic relaxation.

### Thermodynamic structural evolution of crystallites and amorphous regions of spiral fibre muscles during multimodal actuation

To investigate the mechanism of twist preservation by thermodynamic modulation, we next investigated the changes in the crystallinity and the sizes of crystal and amorphous regions in the nylon 6 fibres during thermal annealing (Supplementary Note 4.7). The crystallinity was measured by differential scanning calorimetry (DSC) and XRD. The crystallinity obtained by DSC (40%) was measured as the ratio of the melting enthalpy of the nylon 6 fibre to that of the same mass of fully crystalline nylon 6 (190 J g^−1^) (Fig. S19) [28], and that obtained by XRD (60%) was measured as the ratio of the integrated diffraction intensity of the crystalline regions to the sum of those of the crystalline and amorphous regions [29]. The difference in crystallinity values for DSC and XRD measurements should arise from the different coefficients of enthalpy-to-mass conversion and diffraction-intensity-to-mass conversion for crystalline and amorphous regions. Both measurements showed unchanged crystallinity with thermal annealing up to 180°C for both twisted and non-twisted nylon 6 fibres (Fig. 2a, Figs S15b and S20). In addition, the percentages of α and γ phases in the crystallites were also not affected by twist insertion (Fig. S15b). Therefore, the thermal setting should not originate from new crystal formation in this study.

The nylon 6 fibre contains periodic nanoscale lamellar crystals interconnected by amorphous regions in the fibre axial direction (Fig. S21) [30]. We next characterized the dependence of the sizes of crystals and amorphous regions on thermal annealing (Supplementary Note 4.8). The projection of the periodic lengths in the fibre length direction of the lamellar crystals (*l*_c_) and the amorphous region (*l*_a_), and the sum of these two lengths (*l*_p_, called the long period), can be obtained from the meridional integration of the 2D SAXS patterns corresponding to the fibre length direction and inverse Fourier transformation (Supplementary Note 3.3 and Fig. S22) [31]. Twist insertion in a certain twist range negligibly changed the lengths of the lamellar crystals and the amorphous region while decreasing their projected lengths in the fibre length direction (Supplementary Note 4.9). Thermal annealing (from 25 to 180°C) resulted in monotonic increments of *l*_a_ (from 3.25 to 4.16 nm) and *l*_c_ (from 2.5 to 2.74 nm). After thermal annealing, increased scattering intensity was observed (Fig. 2b, inset), which should originate from the increased electron-density difference between the crystalline and amorphous regions [32]. The above results indicate reorganization of crystallites and amorphous regions during thermal annealing, which should originate from the morphology change of chain segments in the amorphous regions. This is because partial or full shape fixation occurred in the temperature range observed from 120 to 180°C, which is much lower than the melting point (223°C) (Table S2).

Based on the above results, we analysed the structural evolution of twisted fibre artificial muscles induced by thermodynamic-twist coupling (Fig. 2e)and Supplementary Note 4.10). Fibre twisting resulted in spiral molecular chains and torsional stress. Thermal annealing of the tethered twisted fibre resulted in reorganization and stress relaxation of some of the chain segments in the amorphous region, which counterbalanced the relaxation of the molecular chain segments with stress remaining to fix the shape of the twisted fibres. Higher annealing temperatures resulted in more relaxed chain segments, better fixation of the twisted fibre muscles (Fig. 1e)and Fig. S12) and consequently decreased actuation stress of the obtained artificial muscle (Fig. S23). Such actuation occurred with a continuous temperature change because the thermal expansion of the fibre was amplified by the spiral architecture. When the actuation temperature is far lower than the thermal annealing temperature, reversible actuation is observed because no additional relaxation of the chain segments occurs. When the actuation temperature is increased to cause reorganization and stress relaxation of additional chain segments, the artificial muscle cannot recover to the initial length after the actuation cycle. Complete relaxation of the stress-containing chain segments at a sufficiently high actuation temperature causes irreversible actuation (Fig. 2e). The multimodal artificial muscles prepared by this thermodynamic coupling strategy showed the following two characteristics: the shape modulation can occur over a very large continuous temperature range, and the actuation can occur by volume expansion under a continuous temperature change. Consequently, this is different from the shape memory effect, where actuation can only occur when the temperature reaches the critical temperature for memorizing the polymer shape [33].

### Modulation of the morphology and actuation performance by thermodynamic-twist coupling

**Figure 3.**
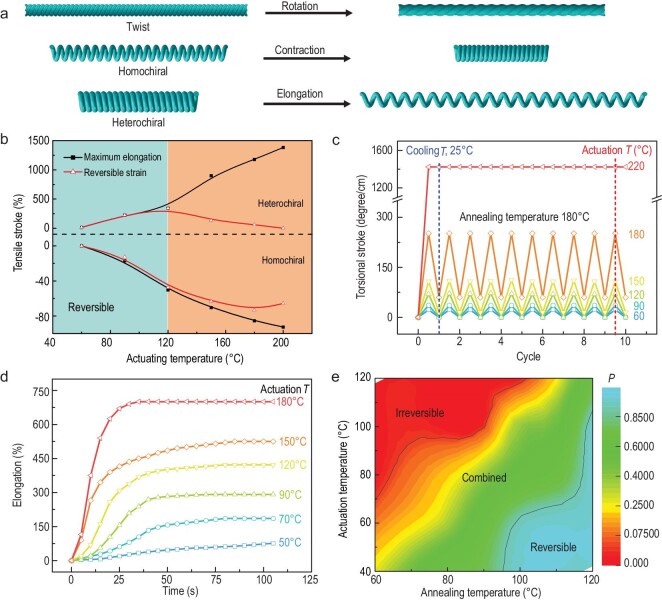
Actuator performance, different types of deformation and different polymer fibres with reversibility modulation. (a) Schematic illustration of torsional, contractile and elongational deformations realized by twisted, homochiral coil and heterochiral coil fibre muscles. (b) Maximum and reversible strokes for torsionally non-tethered homochiral and heterochiral coil nylon 6 fibre muscles at different actuation temperatures, with the annealing temperature fixed at 180°C. (c) Torsional strain as a function of the number of heating/cooling cycles for twisted, non-coiled nylon 6 fibre muscles that are not torsionally tethered during actuation. The twist density was 6.0 turns cm^−1^, and the 0.4-mm-diameter fibre muscles were annealed at 180°C for 1 h while torsionally tethered. (d) Elongation as a function of time for heterochiral coil, 180°C-annealed nylon 6 fibre muscles at different actuation temperatures. For (b) and (d), the diameter of the pristine fibre was 0.45 mm, with a twist density of 5.42 turns cm^−1^ and a spring index of 6.6. (e) The reversibility factor as a function of thermal annealing and actuation temperatures for a 0.6-mm-diameter polyethylene fibre muscle. The inserted twist density was 3.75 turns cm^−1^, and the spring index was 6.0.

We also realized morphological modulation of artificial muscles by employing reversibility control for different types of actuation (contractile and torsional) and for different widely used polymer fibres (PE and nylon 6, 6). Similar to the heterochiral coil muscles, homochiral nylon 6 coil muscles were fabricated using the same handedness of twist insertion and mandrel coiling (Fig. 3a). In contrast to the heterochiral coil muscles, these homochiral coil muscles contracted upon heating due to a twist-release-induced torque that pulled the coils together [23]. Without torsional tethering, the homochiral coil muscle prepared by thermal annealing at 180°C showed a reversible actuation mode when actuated below 120°C and showed combined irreversible and reversible actuation modes when actuated between 120 and 200°C. The maximum contractile strain (92.6%) was obtained by actuation at 200°C, with a reversible stroke of 65.7% (Fig. 3b). We further annealed a twisted, non-coiled nylon 6 muscle at 180°C for 1 h. This fibre muscle untwisted upon heating and showed a reversible actuation mode (below 120°C), an irreversible actuation mode (at 220°C) and combined irreversible and reversible actuation modes (at 180°C), as shown in Fig. 3c.

Using the heterochiral coiled 180°C-annealed nylon 6 fibre muscle again as an example, the actuation strain reached a plateau within 100 s when the actuation temperature was higher than 70°C. Increasing the actuation temperature resulted in higher elongation and a faster actuation speed (Fig. 3d). The actuation speed is expected to increase as a result of decreasing the fibre diameter (thereby increasing the specific surface area of the fibre), using a fibre with a high thermal diffusivity and a low actuation temperature to decrease the ramping time (e.g. PE versus nylon), and increasing the interfacial heat flow by using a fluidic heating media. Note that the maximum reversible stroke remained almost constant (∼300%) when increasing the heating rate from 5 to 40°C min^−1^, showing no dependence of the final stroke on the heating rate (Fig. S5). The actuation performance is dependent on the temperature-relevant material properties (e.g. thermal expansion coefficient and modulus). Similar heterochiral artificial muscles with morphological modulation can also be obtained for different types of polymer fibres, such as PE and nylon 6, 6, by tuning the thermal annealing and actuation temperatures (Fig. 3e, Figs S6–9).

Below, we show that the actuation performance of the above prepared fibre artificial muscles can be delicately adjusted by changing the fabrication and actuation parameters, including the twist density, spring index (coil diameter divided by fibre diameter) and initial coil length, using a heterochiral coil as an example. A mandrel-coiled nylon 6 fibre without twist insertion, thermally annealed at 180°C, showed a relatively small reversible actuation stroke (10.3%) at 150°C. With increasing twist density, both the irreversible and reversible actuation strokes monotonically increased (Fig. S10a). As a control, non-twisted nylon 6 fibres were bent or stretched and thermally annealed at different temperatures. Only irreversible actuation was observed in non-twisted samples at different actuation temperatures (Fig. S11), indicating that the spiral architecture is necessary to obtain a large reversible actuation stroke by magnifying the length change of the fibre muscle (Fig. S11c and d).

This indicates that twist insertion plays a key role in amplifying the actuation strain for this artificial muscle with multimodal actuation. Mandrel-coiled nylon 6 muscles with different spring indices (annealed at 180°C) were actuated at 120, 150 and 180°C. Although a large spring index amplified the muscle stroke, the reversible stroke for actuation of the coiled muscle at 180°C was independent of the spring index because the reversibility factor (fraction of reversible stroke) dramatically decreased as the actuation temperature increased to 180°C (Fig. S10b). Another important point is that compression of the coil during thermal annealing, to provide a shorter coil length, resulted in a larger actuation strain for heterochiral coil muscles (Fig. S10c). These three independent parameters were selected because they can be easily measured and adjusted during the fabrication process. However, a dimensionless parameter (*K*, which is the product of the square of the spring index and the twist density divided by the unit coil length) can be used to quantitatively evaluate the performance by making a few approximations (Supplementary Note 2) [27]. The dependence of the reversible elongation on the spring index simulated using this parameter was consistent with the measured data (Fig. S10d).

### Applications for artificial muscles with different reversibility and morphological modulations

**Figure 4.**
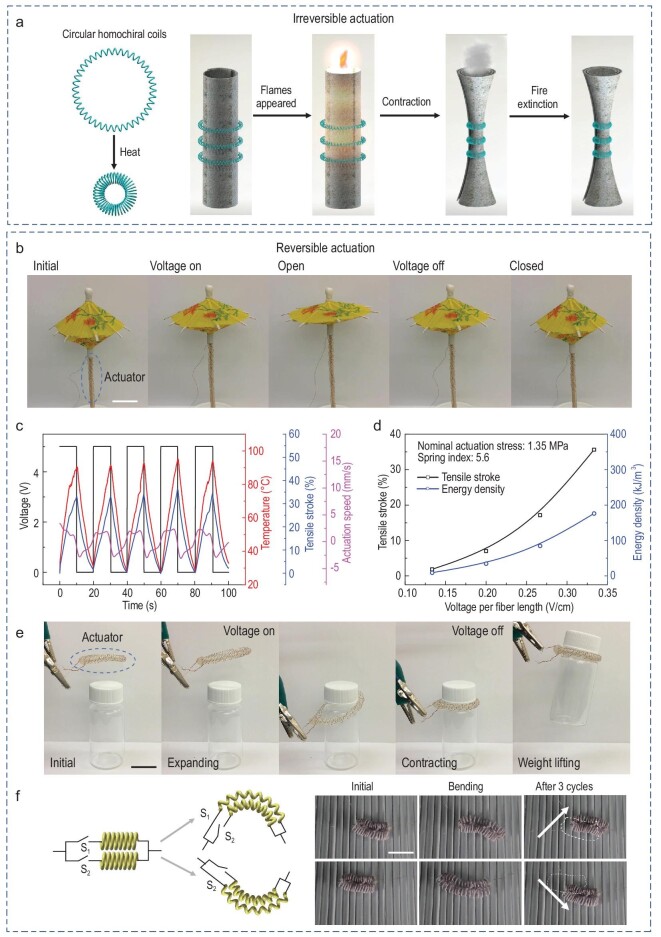
Demonstrations using coiled nylon 6 fibre muscles with irreversible and reversible actuation. (a) Circular homochiral double-coil muscles were used for fire extinction via heat-induced contraction to inhibit the air supply. (b) A coiled heterochiral muscle was used for the electrically driven opening of an umbrella. (c) Actuation performance and (d) calculated energy density of heterochiral coils that were actuated by electrical heating. The spring index was 5.6 and the actuation load was 1.35 MPa for (c) to (d), to meet the conditions in (b) and Movie S6. (e) An electrically driven gripper used to pick up an object. (f) Locomotive soft robot made of two independently controlled coiled heterochiral muscles aligned in parallel, which can control the direction of locomotion. The scale bars in (b), (e) and (f) are 2 cm.

Twist-containing fibre muscles with multimodal actuation, combined with different types of deformation, enable various possible applications. For example, the heating of an assembly of heterochiral coiled nylon 6 muscles can mimic a flower blooming (Movie S1). A heterochiral coiled nylon 6 muscle was wrapped around a mandrel to form a double-coil muscle and tied on parallel steel rods to set the shape. Thermal expansion of the coiled nylon 6 muscle resulted in radial expansion of the double-coil muscle. Irreversible actuation of this muscle enabled different applications. For example, a double-coil muscle carrying epoxy resin can be inserted into a hole-containing tube. Upon heating, the double-coil muscle radially expands, allowing the epoxy resin to contact the broken site and cure to repair the tube (Fig. S24 and Movie S2). It can be used as a catheter, which can expand at the right position after passing through the narrow opening. Upon heating, such a double-coil muscle can also be used to inflate a soft tube (Movie S3). Similarly, a double-coil muscle made from coiled homochiral nylon 6 contracts in the radial direction upon heating. Such a homochiral double-coil muscle wrapping a fire-resistant film can be used to extinguish a fire by removing or limiting its oxygen supply (Fig. 4a)and Movie S4). A homochiral coil muscle can be used to release the liquid encapsulated by a fragile thin film upon heating (Movie S5). Fibre artificial muscles with reversible actuation enable the following applications with reversible deformations: electrically driven umbrellas, roofs, grippers and soft locomotive robots based on heterochiral coils (Fig. 4b–f and Movies S6–10). The peak temperature and actuation speed reached 90°C and 2.5 mm s^−1^, respectively, when a 0.33 V cm^−1^ voltage was applied (Fig. 4c). The tensile stroke and energy density can be adjusted by changing the electrical input for such electrically driven mandrel-coiled muscles, which showed giant tensile strokes (at the cost of energy density) compared with self-coiled muscles (Fig. 4d). For the electrically driven gripper, the maximum load weight can be increased to nearly 1 kg by decreasing the spring index of the mandrel-coiled fibres (Fig. S25a). As another example, two independently actuated heterochiral coil muscles can be assembled in parallel to control the forward movement of a robot (Fig. 4f)and Movie S10). The bidirectional and stepped movements were quantitatively studied and are illustrated in Fig. S25b. A heterochiral muscle can reversibly change its length in response to a temperature change; after an abrupt temperature change, it transforms into another muscle with a different length at room temperature (Movie S11). This effect is also applicable to tuning the length of artificial muscles used in morphing robots and morphing aircraft.

We then investigated the work capacity of these artificial muscles. For an optimized tensile load, the reversible mechanical output and instantaneous power increased for higher actuation temperatures, providing values of 2.1 J g^−1^ for contractile work and 2.5 kW kg^−1^ for contractile peak power when using air heating in an oven for actuation at a temperature of 210°C (Fig. S26a). The mechanical output work and instantaneous power increased with tensile load until muscle fracture (Fig. S26b). The tensile stroke increased to a maximum value and then decreased with increasing tensile load (Fig. S26c). The work capacity and instantaneous power shown in Fig. S26a–c were calculated based on the reversible actuation strokes. During mechanical loading, we observed the first cycle of actuation at different actuation temperatures. The first-cycle work increased with increasing temperature, exhibiting elongation for actuation temperatures lower than 120°C and contraction above 120°C (Fig. S26d). For an actuation temperature of 180°C, the first-cycle work increased to a maximum value and then decreased with increasing tensile load, providing elongation for a loading stress above ∼35 MPa (Fig. S26e). To summarize, the performance metrics of the fibre muscle in this work were compared with those in other reports [2,20,23,34,35], as shown in Fig. S26f and Table S1. The energy efficiency (∼0.1%) might be improved by further increasing the actuation speed using the methods mentioned above so that heat dissipation can be decreased. In addition to the actuation temperature and tensile stress, the geometrical parameters of the fibre muscle also affect the work capacity. For example, with an increase in the spring index, the actuation stroke increased, and the work capacity decreased (Fig. S27).

## CONCLUSIONS

In summary, a thermodynamic-twist coupling strategy was developed to modulate the morphology of artificial muscles by controlling the actuation reversibility to obtain a new muscle. This was realized through the indispensable combination of deformation amplification by the spiral topology and thermodynamic relaxation of the molecular chains. The muscle morphology can be continuously modulated over a wide temperature range for transformation into a new muscle, which exhibits actuation by volume expansion via a continuous temperature change. Such a thermodynamic-twist coupling strategy produced artificial muscles exhibiting powerful actuation performances (contractive work capacity and power density of 2.1 J g^−1^ and 2.5 kW kg^−1^, respectively) and is applicable to different actuation modes, such as contraction, elongation and rotation. This unique design could provide new opportunities for the development of soft robots, morphing aircraft and environmentally adaptive intelligent devices. The spiral amplification and thermodynamic modulation are also applicable to other functional materials with optical, electrical and magnetic properties, with regard to developing novel self-modulating smart devices. The thermodynamic-twist coupling could inspire new research discoveries in other fields, e.g. polymer physics, fibre crystallography and fibre mechanics.

## MATERIALS AND METHODS

### Preparation of twist-containing fibre muscles

The polymer fibres evaluated for artificial muscles with multimodal actuation were nylon 6 fishing line (STRONG & STRETCHY, Model 0.2, 0.4, 0.43, 0.45 from Crystal String), nylon 6, 6 monofilament (0.4 mm in diameter, from Coats and Clark Co. Ltd.) and PE monofilament (0.6 mm in diameter, from Freshwater Shark Co. Ltd.).

To prepare twist-containing fibre muscles, twist was inserted isobarically (under a constant load). A 42-step servomotor (Model 42BYGH40-1704A, Huatian Technology Co. Ltd.) was used to obtain a controlled degree of inserted twist. For twist insertion, the top end of the fibre was connected to the motor, and the bottom end was loaded with a weight and torsionally tethered to prevent rotation of the weight.

To obtain a heterochiral coiled muscle, a twisted fibre was wrapped around a mandrel, keeping the handedness of fibre coiling opposite to the handedness of fibre twist. The heterochiral coil muscle was fully tethered at both ends (with respect to both twist and length change) and thermally annealed at a selected temperature for 1 h in vacuum to fix the shape. The coiled muscle on the mandrel was compressed to minimize the coil pitch (the distance between neighbouring coils) before thermal annealing. This allowed us to obtain a very large elongational strain during actuation. Homochiral coiled muscles were obtained by a similar approach, but using the same handedness of fibre twist and fibre coiling. In most previous work, homochiral muscles were made quite differently (in order to maximize the gravimetric work capacity of the muscle), by inserting twist into a fibre under a constant tensile load until the fibre self-coiled—thereby avoiding mandrel coiling. The pitch distance was maximized by increasing the distance between the two ends of the coiled muscle on the mandrel before thermal annealing. This allowed us to obtain a very large contractile strain during muscle actuation, but reduced the load-lifting capability of the muscle. Twisted, non-coiled fibre muscles were obtained by the following steps. The fibre was isobarically twisted and taped to a steel rod by tethering both ends to avoid twist release during annealing. Then, the fibres were thermally annealed at different temperatures to fix the shape. As a control experiment, it was demonstrated that a non-twisted fibre can be folded in the middle and thermally annealed to fix the shape (Fig. S11). After thermal annealing, the tethering was removed for the coiled heterochiral muscle, coiled homochiral muscle and twist-containing, non-coiled fibre muscle. Unless otherwise indicated, these fibre muscles were actuated free of load at different temperatures to realize multimodal actuation. An infrared (IR) thermometer (FLIR T440) was used to obtain thermal IR images and temperatures. However, for more accurate control of actuation temperature, actuation was conducted in a temperature-controlled oven.

## Supplementary Material

nwac196_Supplemental_Files
