# Supplementary material for: Morphology modulation of artificial muscles by thermodynamic-twist coupling
Source: Natl Sci Rev. 2022 Sep 22;10(1):nwac196. doi: 10.1093/nsr/nwac196 (PMC9843299; doi:10.1093/nsr/nwac196)
Supplement: nwac196_Supplemental_Files [file nwac196_supplemental_files.zip › Supplementary data.pdf]

SUPPLEMENTARY INFORMATION

Morphology Modulation of Artificial Muscles by Thermodynamic-Twist Coupling

Xiaoyu Hu[1,†], Jiatian Li[1,†], Sitong Li[1,†], Guanghao Zhang[1], Run Wang[1], Zhongsheng Liu[1], Mengmeng Chen[1], Wenqian He[1], Kaiqing Yu[1], Wenzhong Zhai[1], Weiqiang Zhao[1], Abdul Qadeer Khan[1], Shaoli Fang[2], Ray H. Baughman[2], Xiang Zhou[3,*], Zunfeng Liu[1,*]

[1]State Key Laboratory of Medicinal Chemical Biology, College of Chemistry and College of Pharmacy, Key Laboratory of Functional Polymer Materials, Frontiers Science Center for New Organic Matter, Nankai University, Tianjin 300071, China
[2]Alan G. MacDiarmid NanoTech Institute, University of Texas at Dallas, Richardson, TX 75080, USA
[3]Department of Science, China Pharmaceutical University, Nanjing 211198, China.

Corresponding authors' emails: liuzunfeng@nankai.edu.cn; zhouxiang@cpu.edu.cn

This PDF file includes:

Supplementary Text (Pages 2–13)
Figures S1 to S31 (Pages 14–30)
Tables S1 to S2 (Pages 31)
Captions for movies S1 to S11(Pages 32–34)
References for SI reference citations (Pages 34-35)

Other supplementary materials for this manuscript include the following:

Movies S1 to S11

Supplementary Note 1. Characterizations Methods

Two-dimensional wide-angle X-ray scattering (2D WAXS) experiments were performed at a sample-to-detector distance of 100 mm using CuKα radiation (λ = 1.54 Å) by deploying a D8 Discover system (Bruker, Germany) equipped with a 2D HI-Star detector and a 1.6 kW generator. Parallel nylon 6 fibers were assembled, using a video microscope to ensure that the X-ray beam irradiated the centers of the fibers. The image acquisition time was 600 s. One-dimensional wide-angle X-ray scattering (1D WAXS) experiments were performed using CuKα radiation and a 2θ scanning rate of 5 ° min^{-1} on a Rigaku Smart Lab system (Japan) equipped with a 3.0 kW generator. Small-angle X-ray scattering (SAXS) was performed on a Xeuss 2.0 system (Xenocs SA, France) equipped with a 2D detector (600×600 pixels, pixel size: 172×172 μm^2) using CuKα radiation and a sample-to-detector distance of 1191 mm. The image acquisition time was 1000 s for SAXS. All WAXS and SAXS measurements were conducted at room temperature, and the patterns were corrected for air scattering, background scattering, and beam fluctuations.

Differential scanning calorimetry (DSC) measurements were carried out on a DSC 204 system (Netzsch Instruments) at a heating rate of 5 ℃ min^{-1} under a N_2 atmosphere. The samples were dried overnight in a desiccator (containing silica drying beads) and tightly packaged before testing.

Supplementary Note 2. Performance of heterochiral actuators by using a single dimensionless parameter that involves the spring index, initial coil length and twist density.

Generally, the elongation of heterochiral actuators is affected by the thermal expansion coefficient of the polymer, the annealing temperature, the actuation temperature, and geometrical parameters. These include the fiber diameter (d), mean coil diameter (D), coil length (L), number of coils (N), spring index (C, $C=D/d$), unit coil length (p, $p=L/N$), inserted twist (n), and twist density (T).

For a twisted, non-coiled fiber, the twist change (Δn) during actuation can be written as Eqn.1.1 (derived from ref. 1).

$$\Delta n=\left(\frac{\Delta\lambda}{\lambda}\frac{1}{\cos^2\alpha_f}-\frac{\Delta d}{d}-\frac{\Delta l}{l}\tan^2\alpha_f\right)n \qquad (1.1),$$

Where λ is the length of the helically oriented polymer chains and l is the projected length of the helical chains in the axial direction. The twist-produced bias angle of these chains is $\alpha_f = \tan^{-1}(\pi dT)$. $\Delta\lambda$ and Δl are the temperature-induced changes in the length of the polymer chains and their projection on the axial direction, respectively. Δd is the change of fibre diameter during actuation.

Here, $T=n/l$, and $\Delta T=\Delta n/l$, where ΔT is the twist density change during actuation, Eqn.1.1 can be written as Eqn.1.2

$$\Delta T=\left(\frac{\Delta\lambda}{\lambda}\frac{1}{\cos^2\alpha_f}-\frac{\Delta d}{d}-\frac{\Delta l}{l}\tan^2\alpha_f\right)T \qquad (1.2).$$

Here λ is considered to be constant during actuation,[2] and the thermal-expansion-produced length change in the fibre direction is assumed to be negligible. Then Eqn. 1.2 can be rewritten as

$$\Delta T=-\frac{\Delta d}{d}T \qquad (1.3).$$

Here, the thermal expansion coefficient of the polymer fibre in the radial direction (α_d) is assumed to be constant in the temperature range studied. Then, Eqn. 1.3 can be expressed as:

$$\Delta T=-\alpha_d T\Delta\theta \qquad (1.4),$$

where $\Delta\theta$ is the temperature change during actuation. For a coiled fibre, the length change ratio and the twist loss during actuation can be expressed as Eqn.1.5 (derived from ref. 1).

$$\frac{\Delta L}{L}=\frac{l^2}{NL}\Delta T \qquad (1.5),$$

where ΔL is the change in coil length during actuation. Considering the coil diameter can also be approximated by $D=l/(\pi N)$, when the spring index is large and the coils before actuation are nearly contacting, Eqn. 1.5 can be rewritten as

$$\frac{\Delta L}{L}=-\pi^2\alpha_d\Delta\theta d^2\cdot\frac{C^2T}{p} \qquad (1.6).$$

When $\alpha_d\Delta\theta d^2$ is approximately constant, $\Delta L/L$ is proportional to K, which is C^2T/p. This is demonstrated for the data in Fig. S10b, where T/p is constant, since in this case $\Delta L/L$ is proportional to C^2, which is the square of spring index.

Supplementary Note 3. Calculation of the crystallinity, degree of polymer crystal orientation, and periodic lengths of lamellae crystals and amorphous regions

3.1 Calculation of crystallinity using WAXS analysis

The crystalline component of the nylon 6 fibres contains both crystalline α and γ phases. The crystallinity and contents of α phase and γ phase was calculated by multi-peak fitting of 1D WAXS and areal calculation of the corresponding peaks, using CuKα radiation diffraction angles (2θ) ranging from 5° to 50°. The details of peak fitting are as follows. The peak fitting was conducted using Peakfit software. The amorphous peak center position (halo center) was set at a 2θ of 21.4°, according to the literature [3], and the amorphous peak ends should overlap the measured 1D WAXS curve. The peak

center positions for the α and γ phases were obtained directly from the recorded 1D WAXS curve. By using the centers for the amorphous and crystalline peaks, the fitting peak curves were obtained by using the function of Pearson VII, as shown in Fig. S20a. The crystallinity and the contents of α phase and γ phase were calculated using the ratios of the areas of the crystalline peaks (the α phase peaks, and the γ phase peaks, respectively) to the sum of the areas of crystalline and amorphous peaks, as shown in Fig. S20b.

3.2 Calculation of degree of orientation of crystals using WAXS analysis

The degree of crystal orientation was used to evaluate the retained twist in the fibre muscle. The detailed calculations were as follows. First, an azimuthal curve was obtained by integration of diffraction intensity in the 2D WAXS pattern over the azimuthal angle using the software of the GADDS (general area detector diffraction system) for a diffraction angle ($2\theta_d$) ranging from 20.5 ° to 22 °, as shown in Fig. S15. Two identical peaks were observed in the azimuthal curve because of the symmetric diffraction pattern in 2D WAXS. Then the full width at half maximum (FWHM) of one peak was obtained, and called θ_h. The degree of orientation of crystals (DOC) was calculated using the following equation

$$DOC=(180^\circ-\theta_h)/180^\circ\times100\% \quad (2.1).$$

3.3 Calculation of the periodic lengths of lamellae crystals and amorphous regions in 2D SAXS analysis

The periodic lengths of the lamellar crystals (l_c) and the amorphous region (l_a), and the sum of these two lengths (l_p, called the long period), can be obtained from the 2D SAXS patterns, by using meridian integration and inverse Fourier transformation, according to the literature [4]. The calculation details are as follows. First, radial integration along the meridian from the 2D SAXS pattern using a width of 10 pixels gives the curve of scattering intensity $I(q)$ as a function of scattering vector (q), where $q = 4\pi\sin\theta_d/\lambda$ and λ is the wavelength (1.54 Å) (Fig. S22a, inset). Second, inverse Fourier transformation was carried out on the $I(q)$-q curve to obtain a one-dimensional correlation function $\gamma(Z)$-Z, which is defined by equation (2.2):

$$\gamma(Z)=\frac{1}{Q}\int_0^\infty I(q)q^2\cos(qZ)\,dq \quad (2.2),$$

where Z is the length along the fibre axis, and Q is the total scattering, which is calculated by equation (2.3)

$$Q = \int_0^{\infty} I(q) q^2 \mathrm{d}q \quad (2.3).$$

The total scattering Q and the $\gamma(Z)$ curves were obtained by use of SasView software. The calculation details are as follows (Fig. S22). First, a $I(q)q^2$-q curve was obtained from the $I(q)$-q curve using Lorentz calibration. Then integration of the $I(q)q^2$-q curve by extrapolation of the low-q and high-q area by use of the Guinier function and the Porod function gave the total scattering Q. Then the γ(Z) curves were calculated from equation (2.2). The l_c, l_a, and l_p can be obtained from the γ(Z) curves. The l_c was the Z value by extrapolation of the linear region of the γ(Z) curve before the first minimum (negative value), to the minimum value of γ(Z). The l_p was the Z value of the γ(Z) curve at the first maximum (positive value). The l_a was obtained by $l_a = l_p - l_c$.

3.4 Correlation of scattering intensity with the electron density of crystalline and amorphous regions

We observed an increase in scattering intensity in 2D SAXS with increasing annealing temperature (Fig. 3b in main text). To explain this, we here discuss the correlation of scattering intensity with electron density of crystalline and amorphous regions.

For polymers with lamellar long-term structures, the total scattering (Q) in 2D SAXS as a function of the electron density of crystal region (ρ_c) and amorphous region (ρ_a) can be described by equation (2.4):

$$Q \propto \phi_s(1-\phi_c)\phi_c(\rho_c-\rho_a)^2 \quad (2.4),$$

where ϕ_s is the volume fraction of crystalline regions and $\phi_c = l_c/l_p$ [5].

From the results in Fig. 3a, it can be seen that ϕ_s did not change upon thermal annealing. Fig. S28 shows that the ϕ_c of the non-twisted nylon 6 fibre is negligibly changed by increasing the thermal annealing temperature from 25 to 180 ℃. Therefore, the change in total scattering Q should be mainly ascribed to the electron density difference between crystalline region and amorphous region (ρ_c-ρ_a). From the results in Fig. 3b, it can be concluded that the intensity of the scattering peak of non-twisted nylon 6 fibre increased with increasing thermal annealing temperature, which should originate from an increased electron-density difference between the crystalline region and amorphous region.

3.5 Theoretical calculation of periodic length for crystalline and amorphous regions for twisted fibre muscles

The projection of the periodic length in the fibre length direction for crystalline ($l_{c,T}$), amorphous regions ($l_{a,T}$) and the sum of these two lengths ($l_{p,T}$) for twisted nylon 6 fibre at an inserted twist density T can be theoretically calculated based on the integration of the axial projections of these lengths at zero twist density ($l_{c,0}$; $l_{a,0}$; and $l_{p,0}$). The calculation details are as follows. By isobaric twist insertion into the nylon 6 fibre with a twist density T, the amorphous regions and lamellar crystals form spiral architectures with a bias angle (α_r) at a different radius (r), as shown in Fig. S29. The bias angle can be calculated using equation (2.5)

$$\alpha_r = \tan^{-1}(2\pi r T) \quad (2.5).$$

Using the laminar crystals as an example, for a twisted fibre with a twist density T, the measured $l_{c,T}$ was the projection of the periodic length of lamellar crystals at zero twist ($l_{c,0}$) in the axial direction. Note that the bias angle α_r at different layer radius r is different for a twisted fibre. So, the projected length of $l_{c,T}(r)$ at a layer radius r for a given twist density T should depend on the bias angle α_r, as calculated using equation (2.6)

$$l_{c,T}(r) = l_{c,0} \cdot \cos\alpha_r \quad (2.6),$$

where $l_{c,0}$ is the periodic length of lamellar crystals for non-twisted nylon 6 fibre. The $l_{c,T}$ can be obtained by the integration of $l_{c,T}(r)$ over the layer radius (r) from the axis to the surface of a twisted fibre based on the area fraction of each layer, and it can be calculated by equation (2.7)

$$l_{c,T} = \int_0^{R_T} \frac{2\pi r}{\pi R_T^2} \cdot l_{c,T}(r) \cdot \mathrm{d}r \quad (2.7).$$

By substituting equations (2.5) and (2.6) into equation (2.7), we can obtain equation (2.8)

$$l_{c,T} = l_{c,0} \cdot \frac{\sqrt{(2\pi T R_T)^2+1}-1}{2\pi^2 T^2 R_T^2} \quad (2.8).$$

Similarly, the periodic lengths of the amorphous region and the long period can also be calculated in the same way.

Supplementary Note 4. Microstructural evolution of the nylon 6 fibre muscles during thermal annealing and actuation

To understand the mechanism of the above multi-modal actuation for nylon 6 fibre muscles, we investigated the microstructural evolution of the crystalline and amorphous phases during muscle preparation and after actuation. All X-ray diffraction (XRD) measurements were conducted at room temperature in this and the next sections, to eliminate the effects of thermal expansion. As the artificial muscles were fixed by thermal annealing and the difference between the irreversible and reversible

actuation is whether the coiled muscle length can be recovered after thermal actuation, it is important to study the origin of this annealing-induced shape fixation.

4.1 Shape fixity during thermal annealing

The shape fixity, which is used to evaluate the extent of shape fixation by thermal annealing, is defined as the ratio of the tethered coil length obtained by mandrel coiling to the non-tethered coil length (after 24 hours at room temperature) (Fig. S12a). Thermal annealing of the heterochiral coil muscle below 170 ℃ cannot completely fix the shape, and the coiled muscle will extend at room temperature upon removal of tethering. The coiled muscle's extension at room temperature decreased with increasing annealing temperature. Annealing the coiled muscle at 180 ℃ fixed the coil shape, which showed negligible extension (<1%) in 24 hours at room temperature; and annealing a twisted, non-coiled nylon 6 fibre muscle above 180 ℃ for one hour can also avoid fibre untwisting at room temperature (Fig. S12). Because nylon 6 is a semi-crystalline polymer, we investigated if this shape fixation was due to crystal growth during thermal annealing. For convenience of characterization, the torsional actuation of twisted, non-coiled fibre muscles were used in this and the next sections.

4.2 Length and diameter change during twist insertion

A nylon 6 fibre with length L_0 and radius R_0 was tethered with respect to length during twist insertion and subsequent annealing at 180 ℃ for one hour, and then returned to room temperature while fully tethered. Afterwards, tethering was completely removed at room temperature (so that length changes and partial untwist could occur). Subsequently, the fibre length (L_T) and the fibre diameter (R_T) were measured. Partial untwist did not significantly occur. Fig. S13 shows the dependence of L_T/L_0 and R_T/R_0 on the twist density after annealing at 180 ℃ for one hour.

4.3 Characterization of orientation of crystals for twisted fibres by WAXS

First, the crystal alignment in WAXS was used as an indicator to investigate the selectivity of the multimodal actuation for the twist-containing artificial muscle. The crystal phases in the pristine fibre showed high anisotropy as indicated by the corresponding WAXS patterns, which originated from the melt-spinning and mechanical draw processes used for fibre fabrication. Inserting twist in nylon 6 fibres generated a bias angle between the alignment direction of the microstructures (including crystals and amorphous chains) and the fibre length direction. For example, a longer arc length in the WAXS for the twisted fibre muscle was observed, compared with that for the non-twisted fibre (Fig. 3c, inset, in main text), which was due to the variation in the bias angle of the crystal alignment direction from the fibre surface to the fibre axis (Fig. S14, inset) [6]. The degree of orientation of crystals (DOC), used to

evaluate the retained twist in the fibre, is calculated as (180 °–θ_h)/180 °, where θ_h is the arc angle at the half-maximum integrated diffraction intensity from WAXS patterns (Fig. S15). The DOC increased with time as a non-annealed twisted muscle was left untethered at room temperature (Fig. S16a), indicating untwisting of the crystals and stress relaxation in the twisted muscle. Thermal annealing the tethered twisted nylon 6 muscle decreased this crystal untwisting at room temperature, and negligible untwisting was observed for the twisted muscle having an annealing temperature of 180 ℃ (Fig. S16b). This agrees with results showing that the shape fixity was nearly 100% for twisted and coiled nylon 6 fibre muscles annealed at 180 ℃ (Fig. S12).

4.4 Characterization of orientation of crystals for twisted fibres by SAXS

Arc-shaped scattering patterns were also observed in SAXS for twisted nylon 6 fibre muscles annealed at 180 ℃ (Fig. 3d, inset, in main text). Similar to the WAXS results (Fig. 3c, inset, in main text), this should originate from the variation in the alignment direction of lamellar crystals and amorphous regions from the fibre surface to the fibre core. We then correlated this arc pattern in SAXS with the retained twist density of nylon 6 fibre muscles. The ratio of the sagitta (h) of the inner arc to the corresponding chord length (c), h/c, was plotted as a function of inserted twist for nylon 6 fibre muscles annealed at 180 ℃ (Fig. S17). It can be seen that h/c monotonically increased from 0 to 0.134 as the twist density of the fibre muscle increased from 0 to 6.0 turns cm^{-1}. As the inserted twist can be retained by annealing the nylon 6 fibre muscles at 180 ℃ for one hour, the h/c ratio was used as a twist indicator for investigating multimodal actuation at different actuation temperatures.

4.5 Change of DOC of artificial muscles with different reversibility during actuation

The DOC can serve as an indicator of the retained twist in fibre muscles. We next investigated the change in DOC for the artificial muscle with irreversible and reversible actuation modes and their combinations (Fig. 3c in main text). Inserting 14.0 turns cm^{-1} of twist into a 0.2-mm-diameter nylon 6 fibre, followed by thermal annealing at 180 ℃, provided an artificial muscle with a DOC of 46.1%, compared to 92.5% for non-twisted fibre. The DOC did not increase after torsional actuation at 120 ℃ for five thermal cycles, indicating that the twist retained in the fibre muscle was negligibly changed. This corresponded to the artificial muscle with reversible torsional actuation modes. The DOC increased to 54.9% and 70.1% after muscle actuation at 180 and 210 ℃, respectively, and it did not change in the following cycles of actuations. This indicates that more twist in the fibre muscle was released when the muscle actuates at 210 ℃, compared to actuation at 180 ℃. This corresponded to the irreversible stroke present in the fibre muscles for both irreversible mode when actuating at 210 ℃ and for the fibre muscles

with combined irreversible and reversible mode when actuating at 180 ℃. Similar effects were observed for the fibre at an annealing temperature of 150 ℃ (Fig. S18).

4.6 Change of *h*/*c* of artificial muscles with different reversibility during actuation

The fibre muscle annealed at 180 ℃ (with 6.0 turns cm^{-1} of inserted twist) was subsequently used for thermal actuation. As calculated from the 2D SAXS scattering patterns in Fig. S30, the *h/c* did not change after actuating at 120 ℃ for five cycles, indicating that the retained fibre twist was negligibly changed (Fig. 3d). This corresponded to the artificial muscle with reversible actuation. The *h/c* decreased from 0.134 to 0.085 and 0.056 for the fibre muscle after actuation at 180 and 210 ℃, respectively, and did not change for the following cycles of actuation. This indicates that more twist in the fibre muscle was released when the muscle actuated at 210 ℃ compared to actuation at 180 ℃. This also corresponded to the irreversible strokes present in the artificial muscle with irreversible actuation at 210 ℃ and the artificial muscle with combined irreversible and reversible actuation at 180 ℃ (Fig. 3d in main text).

4.7 Characterization of crystallinity of nylon 6 during thermal annealing

The crystallinity of nylon 6 is the mass percent of the crystalline phase relative to that of the total fibre, which can be indirectly measured by differential scanning calorimetry (DSC) as the ratio of the melting enthalpy of the nylon 6 fibre to that of the same mass of fully crystalline nylon 6 (190 J g^{-1}) [7], as shown in Fig. S19. Fig. 3a in main text shows that the crystallinity remained unchanged (~40%) upon annealing the non-twisted nylon 6 fibre from 25 to 180 ℃, and annealing the twisted nylon 6 fibre muscle (6 turns cm^{-1}) led to negligible crystallinity differences with the non-twisted fibre. As an alternative, the crystallinity of the nylon 6 fibre was also measured by XRD, using the ratio of the integrated diffraction intensity of the crystalline regions to the sum of that for the crystalline and amorphous regions [3]. The crystallinity remained almost constant (~60%) in XRD measurements for non-twisted nylon 6 samples thermally annealed at 60 to 180 ℃ for one hour. The percentages of α and γ phases in the crystalline phase for nylon 6 fibre also remained constant with varying annealing temperature (Fig. 3a, inset in main text, Fig. S20). Both measurements showed the independence of crystallinity on the annealing temperature up to 180 ℃. In addition, the crystallinity and percentages of micro phases were also not affected by the inserted twist in the twist range investigated (Fig. S15b). The difference in crystallinity values for DSC (~40%) and XRD (~60%) measurements might arise from the different coefficients of enthalpy-to-mass conversion and diffraction-intensity-to-mass conversion for crystalline and amorphous regions. Since the above nylon 6 samples for multimodal actuation were

prepared at an annealing temperature ranging from 120 to 180 ℃, shape fixation should not arise from new crystal formation.

4.8 Characterization of sizes of lamellar crystals and amorphous region of nylon 6 fibre by thermal annealing

It has been reported that the nylon 6 fibre contains periodic nanoscale lamellar crystals interconnected by amorphous regions (Fig. S21) [8], as confirmed by the small angle X-ray scattering (SAXS) results in Fig. 3b, all of which were measured at room temperature after the annealing process. The lamellar crystals and the amorphous regions are periodically stacked along the fibre length direction for the non-twisted nylon 6 fibres. By twist insertion into the fibre, there is a bias angle between the alignment direction of the lamellar crystals and the amorphous regions with the fibre length direction. The projection of the periodic lengths in the fibre length direction of the lamellar crystals (l_c) and the amorphous region (l_a) and the sum of these two lengths (l_p, called the long period) can be obtained, from the meridional integration of the two-dimensional SAXS patterns corresponding to the fibre length direction and inverse Fourier transformation (Supporting Note 3.3, Fig. S22) [4]. The above integration of l_c, l_a, and l_p was carried out in the doted rectangular region shown in Fig. S22a. This rectangular region corresponds to the fibre length direction, so that the obtained values of l_c, l_a, and l_p are the projections in the fibre length direction of the periodicities of the lamellar crystals, the amorphous region, and the sum of these two lengths. Note that the shape of the coiled nylon 6 fibre muscle can be partially or fully fixed at an annealing temperature between 120 and 180 ℃, which is much lower than the melting point (223 ℃) (Table S2). The shape fixation should originate from an annealing-induced morphology change of chain segments in the amorphous regions.

The non-twisted nylon 6 fibres were annealed at a temperature up to 210 ℃ for one hour, and two-dimensional SAXS was conducted on these samples. For the fibres with annealing temperatures from 25 to 180 ℃, l_a monotonically increased from 3.52 to 4.16 nm (an 18.2% increase) (Fig. 3b in main text). This indicates that the chain segments in amorphous regions changed their morphology, thus reorganizing the assembly. Note that l_c also increased from 2.50 to 2.74 nm (a 9.6% increase), and the intensity of the scattering peak increased (Fig. 3b, inset in main text), which should originate from the increased electron-density difference between the crystalline and amorphous regions [5]. Therefore, the assembly of the lamellar crystals also changed, due to re-organization of the amorphous polymer chains, although the total amount of crystals did not increase during annealing, as confirmed by the XRD and DSC results in the previous section (Fig. S20 and Fig. 3a in main text).

4.9 Characterization of sizes of lamellar crystals and amorphous region of nylon 6 fibre by twist insertion

The first image of the inset in Fig. 3b shows the SAXS pattern of a pristine nylon fibre. The two scattering spots in the vertical direction indicate periodic inter-stacking of crystal region and amorphous regions along the fibre length direction. The amorphous regions and lamellar crystals form a spiral architecture upon twist insertion in nylon 6 fibre muscles. As discussed in the previous section, the projection of the periodic lengths (l_c, l_a, and l_p) in the fibre length direction for crystalline, amorphous regions, and the sum of these two lengths for twisted nylon 6 fibre muscles, respectively, were obtained by integration of the SAXS intensities corresponding to the fibre length direction. The l_c, l_a, and l_p monotonically decrease with increasing twist density (Fig. S31), which should be correlated to the spiral architecture of the crystalline and amorphous regions. Because l_p decreases with increasing twist density. Inversely, an increasing l_p should correspond to a decreasing twist density for the fibre. Consequently, fibre untwist during actuation should correspond to an increasing l_p. Nylon 6 fibres with different inserted twist were annealed at 180 ℃ for one hour and used for SAXS studies. From the WAXS results (Fig. S16), it was shown that the inserted twist was maintained even after the thermally-set twisted fibres were return to room temperature and completely untethered (which agrees with the observed absence of untwist).

We next theoretically calculated these projected lengths ($l_{c,T}$, $l_{a,T}$, and $l_{p,T}$) in the fibre length direction as a function of the inserted twist density (T), by integration of the axial projections of these lengths at zero twist density ($l_{c,0}$, $l_{a,0}$, and $l_{p,0}$), from the surface of the twisted fibre to its core. We use $l_{c,T}$ as an example for describing calculation details (Supporting Note 3.5). The projected length of $l_{c,T}(r)$ at a layer radius r for a given twist density T should depend on the bias angle α_r. Since the bias angle α_r at layer radius r varies from the fibre surface to the fibre core for a twisted fibre, the $l_{c,T}$ can be obtained by integration of $l_{c,T}(r)$ over the layer radius r from the fibre core to the fibre surface (Eqn. 2.8 in SI). Similarly, the periodic lengths of the amorphous region ($l_{a,T}$) and the long period ($l_{p,T}$) can also be calculated.

The above theoretically calculated projected lengths of crystalline, amorphous region, and sum of these two lengths at different inserted twist agree with the integrated values measured by SAXS, indicating twist insertion for these twist densities does not change these sizes (Fig. S31). The slight deviation of theoretical calculations with experimental measurements of l_p and l_a at high twist density indicates a morphological change for the amorphous region.

4.10 Structural evolution of twisted artificial muscles by thermally switching muscle twist

The mechanism for the shape fixation of twist-containing fibre muscles by thermal annealing is thus suggested as follows. Upon twist insertion, the polymer chains in fibres were deformed, and stress was generated in the polymer chains. Annealing of the tethered twisted fibre resulted in some of the chain segments reorganizing their morphology and releasing internal stress. These stress-released chain segments then served as a counter balance against the stress-containing chain segments and fixed the shape of twist-containing nylon 6 fibre muscles. The length of the amorphous regions increased monotonically with increasing annealing temperature (Fig. 3b in main text), indicating that an increasing reorganization of chain segments occurred during increasing temperature anneal. This agrees with our observations that increasing the annealing temperature resulted in a higher shape fixity of the twisted or coiled nylon 6 muscle (Fig. S12). The above mechanism was further confirmed by our following investigations on microstructural evolution during shape fixation and actuation of twisted fibre muscle by wide angle and small angle X-ray scattering (WAXS and SAXS) measurements.

Based on the above microstructural evolution for fibre muscles during twisting, thermal annealing, and actuation, the twist-containing fibre artificial muscles with multimodal actuation is realized as follows (Fig. 3e in main text). Inserting twist into the polymer fibre results in the lamellar crystalline and amorphous regions forming a spiral structure. If torsional tethering was removed from the twisted fibre muscle, then these regions tended to release some of this twist-induced internal stress at room temperature. Thermally annealing the two-end torsionally tethered, twisted fibre muscle resulted in part of the chain segments in the amorphous regions changing their morphology to release some of the internal stress, even when overall fibre untwist was prohibited. These amorphous chain segments in the fibre muscles served as an internal counter balance that can restrict twist release from the remaining stress-containing microstructures (crystalline and amorphous regions) when tethering was removed from the fibre muscle during actuation. More chain segments can release their internal stress at a higher annealing temperature, which led to a higher shape fixity of the fibre muscle (Fig. S12). This was further supported by the fact that less actuation stress was generated at 90 ℃ for heterochiral coiled PE fibre muscles with increased annealing temperature (Fig. S23).

For the twisted fibre artificial muscle with reversible actuation (e.g., for nylon 6 fibre with an annealing temperature of 180 ℃ and an actuation temperature of 120 ℃), no more stress-containing amorphous chain segments released their internal stress after muscle actuation because the actuation temperature was much lower than the annealing temperature. As the actuation temperature increased to

above or close to the annealing temperature of the artificial muscle, more stress-containing amorphous chain segments in the fibre muscle changed morphology during actuation, resulting in a partially irreversible stroke (combined irreversible and reversible actuation) or a fully irreversible actuation stroke (irreversible actuation) for the fibre muscle, respectively. It should be noted that this mechanism only focused on the selectivity of actuation modes and that thermal expansion combined with spiral architecture provide the important force for actuation [1]. The multi-modal artificial muscles prepared by this thermally twist-switching strategy showed the following two characteristics. One is that the reversibility can be modulated at any temperature between room temperature and the melting temperature; and the actuation can occur at any temperature from room temperature to the melting temperature. Consequently, this is different from the shape memory effect, where the shape deformation can only occur when the temperature reaches the critical temperature for memorizing the polymer shape.

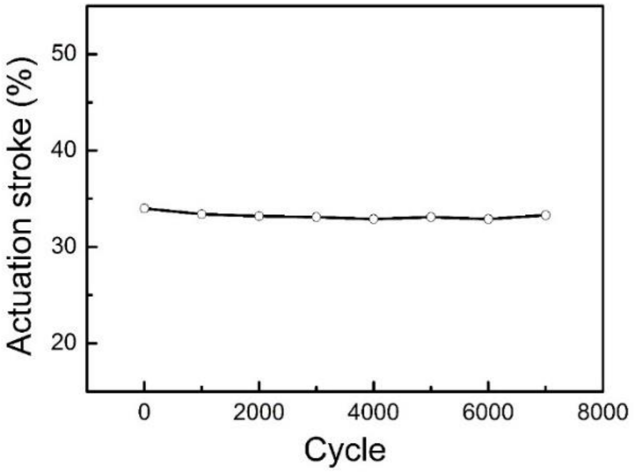

Figure S1. Actuation stroke as a function of the number of heating/cooling cycles for a coiled heterochiral, 0.45-mm-diameter nylon 6 fibre muscle. The nylon 6 fibre and a 0.05-mm-diameter copper wire were plied together, wrapped around a 3-mm-diameter mandrel, and thermally annealed at 180 ℃ for one hour to set the shape. The spring index was 6.6. A 0.05 V cm^{-1} square-wave potential (normalized to wire length) with an on/off frequency of 0.25 Hz was applied.

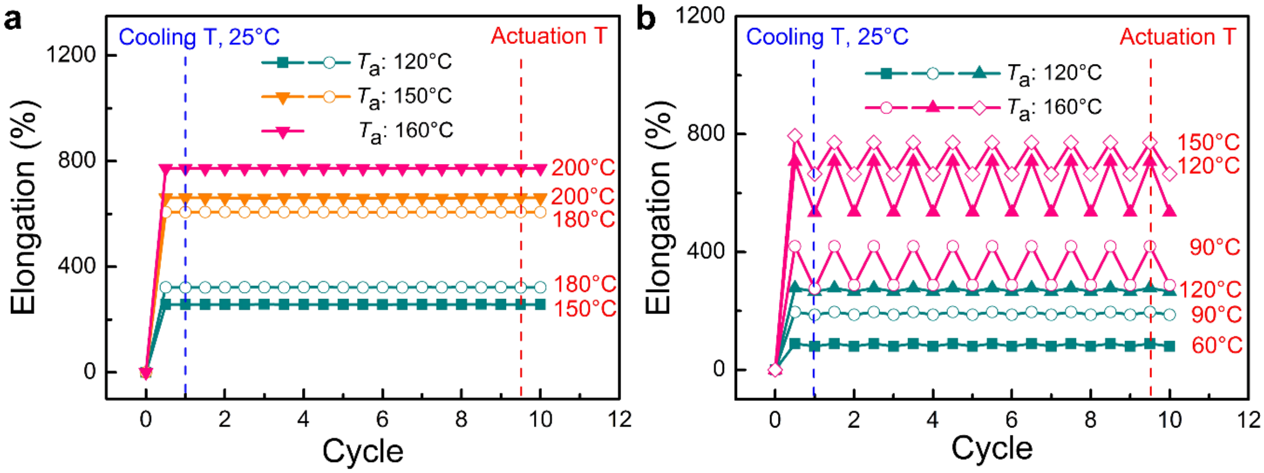

Figure S2. Elongation as a function of number of heating/cooling cycles for coiled heterochiral nylon 6 fibre muscles with (a) irreversible, and (b) combined irreversible and reversible actuation modes, when the thermal annealing temperature was lower than thermal-set temperature. The fibre diameter was 0.45 mm.

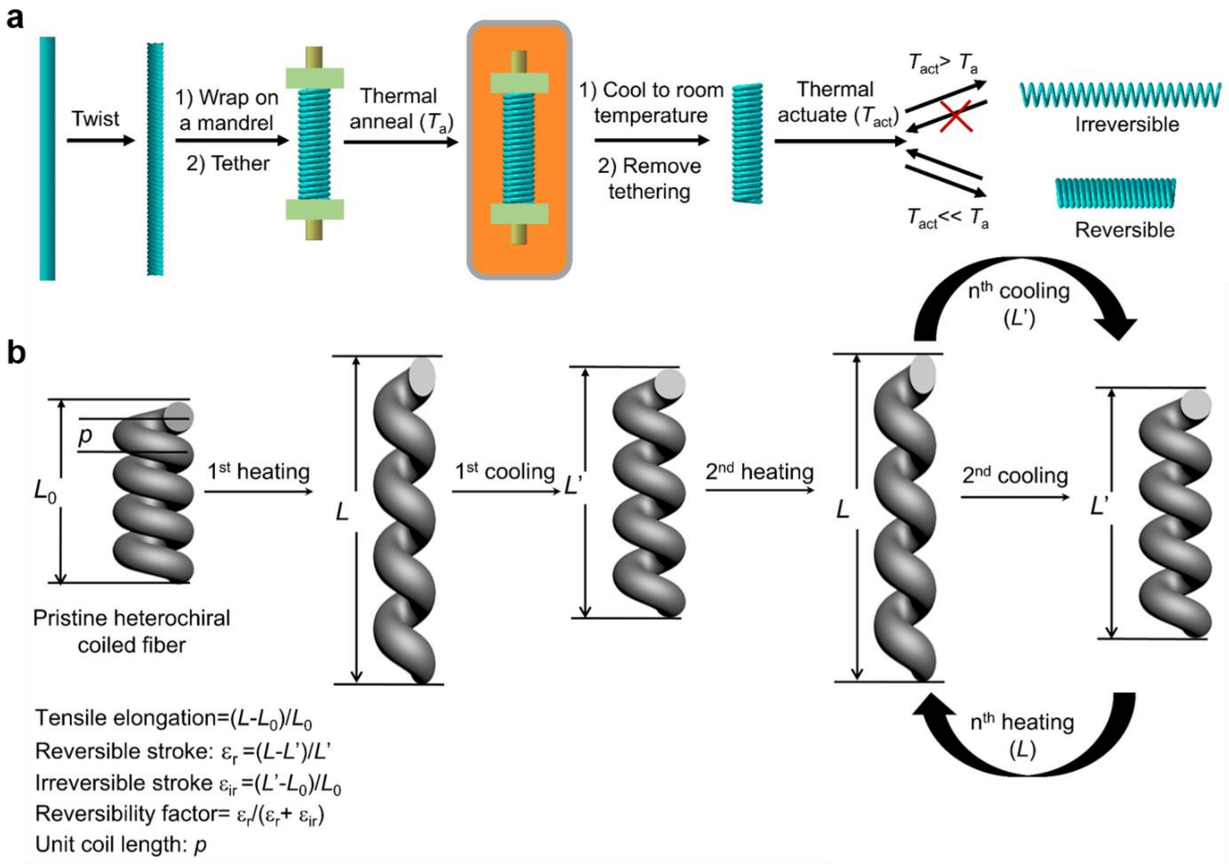

Figure S3. (a) Schematic illustration of the fabrication of heterochiral coiled nylon 6 muscles and their irreversible and reversible actuation during heating. (b) Schematic illustration for calculation of tensile stroke, reversible stroke and irreversible stroke during thermal actuation, using a coiled heterochiral fibre muscle as an example. The function of the steel rod is as follows. For preparation of a heterochiral coil, this nylon 6 fibre was twisted in the S direction and wrapped around a 3-mm-diameter steel rod in the Z direction to form a heterochiral coil. The steel rod serves as the support for the coil, and the coil was tethered on the coil. Then the fixed coil was annealed at 180 ℃ for one hour. The shape of the coil was retained after cooling to room temperature (25 ℃), and then we removed the steel rod to obtain a self-supporting coiled artificial muscle. he function of the metal wire is serving as the support for preparing the coiled artificial muscle. Such self-supporting artificial muscle can actuate by itself by heating, without the need of wire.

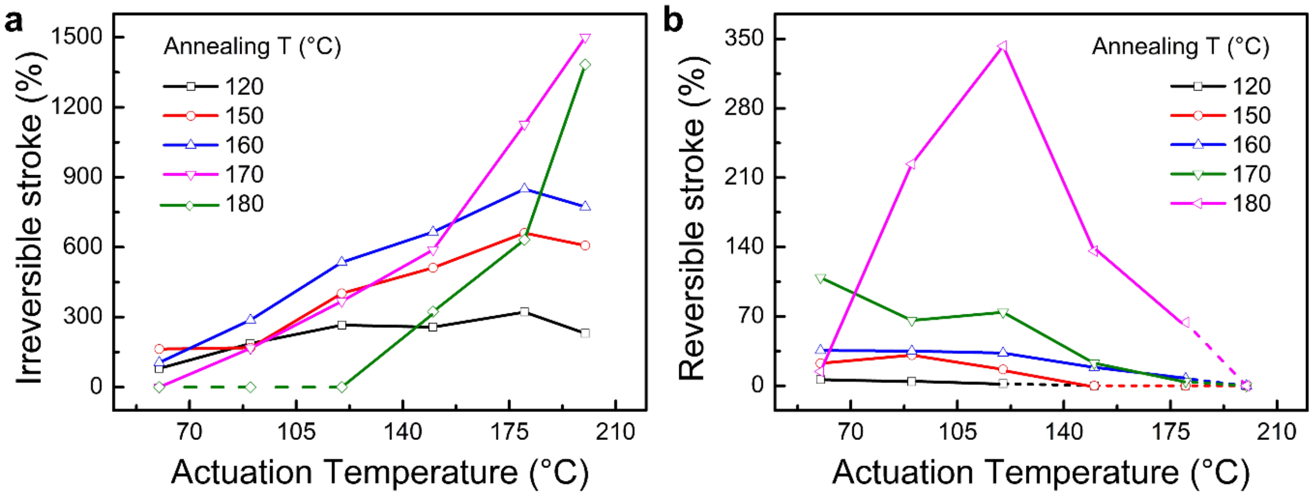

Figure S4. (a) Irreversible and (b) reversible strokes for coiled heterochiral nylon 6 fibre muscles at different actuation temperatures. The twist density was 5.42 turns cm^{-1}, the spring index was 6.6, and the fibre was thermally annealed for one hour.

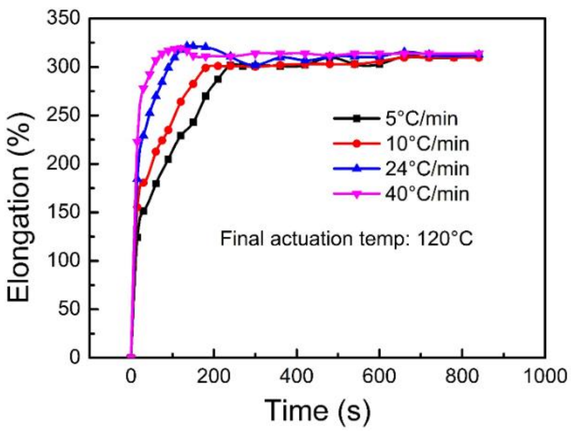

Figure S5. Elongation of heterochiral nylon 6 fibres for different heating rates. The 0.43 mm-diameter-fibre was twisted with density of 5.0 turns cm^{-1} and annealed at 180 ℃ for one hour. The spring index was 5.6. The final actuation temperature was 120 ℃.

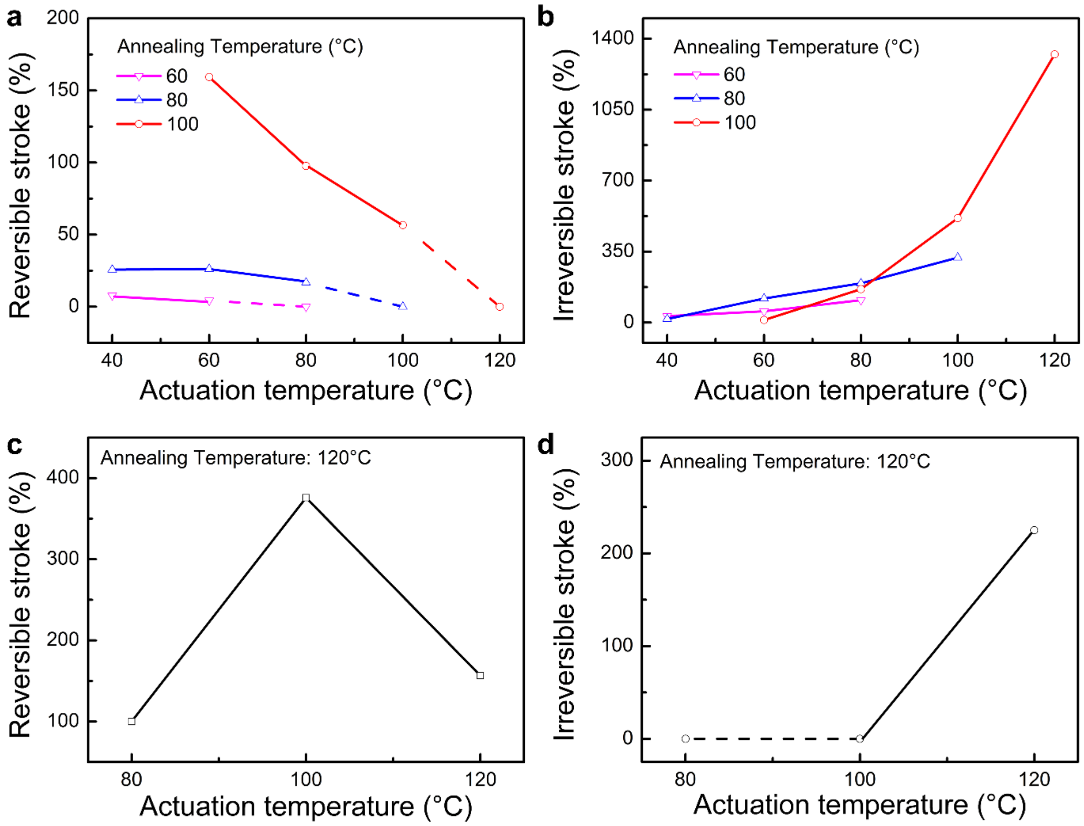

Figure S6. Reversible and irreversible actuation strokes for coiled heterochiral polyethylene fibres. (a) Reversible and (b) irreversible actuation strains for polyethylene fibre muscles that have been annealed at 60, 80, and 100 ℃ for one hour and actuated at different temperatures. (c) Reversible and (d) irreversible actuation strains for polyethylene fibre muscles that have been annealed at 120 ℃ for one hour and actuated at different temperatures. For (a) to (d), the inserted twist was 3.75 turns cm^{-1}, and the spring index was 6.0. The dashed lines indicate that the strain decreased to 0% with increasing actuation temperature.

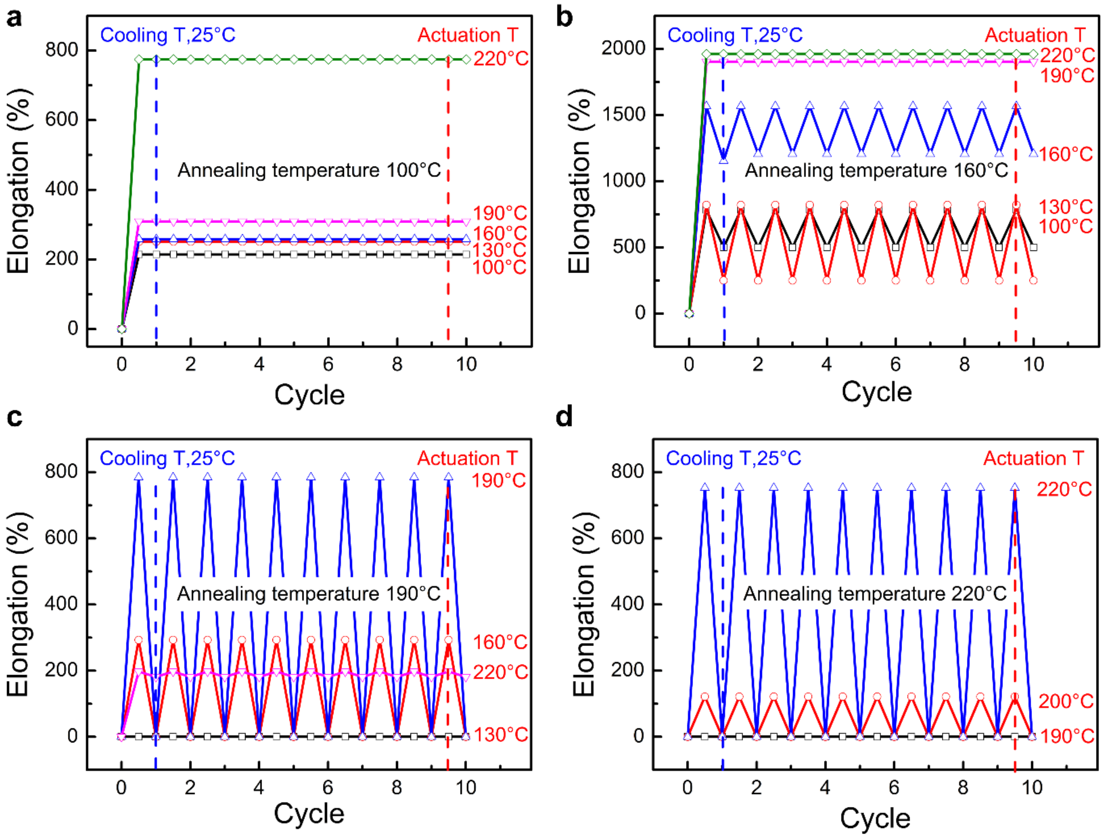

Figure S7. Elongation as a function of the number of heating/cooling cycles for coiled heterochiral nylon 6, 6 fibre muscles with different thermal annealing and actuation temperatures. The twist density was 6.25 turns cm^{-1}, the isobaric load for twist insertion was 15.6 MPa, the fibre diameter was 0.4 mm, the coil spring index was 8.5, and the thermal annealing time was one hour.

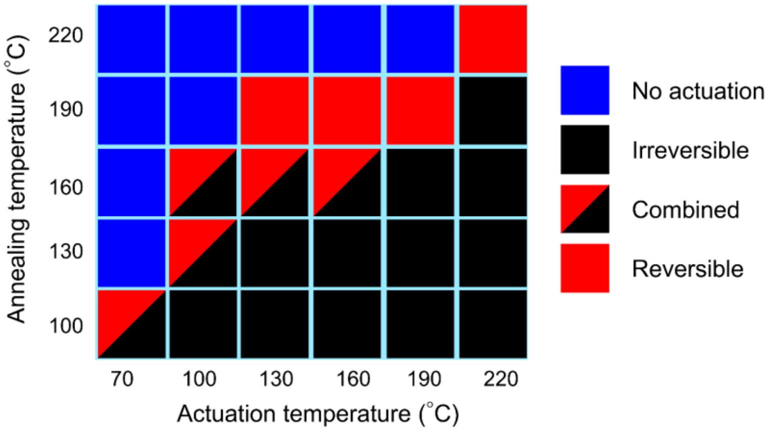

Figure S8. A diagram showing the dependence of multimodal actuation modes (irreversible, reversible, or their combination) for coiled heterochiral nylon 6,6 fibre muscles having different combinations of thermal annealing and actuation temperatures. The twist density was 6.25 turns cm^{-1} and spring index was 8.5. The thermal annealing time was one hour.

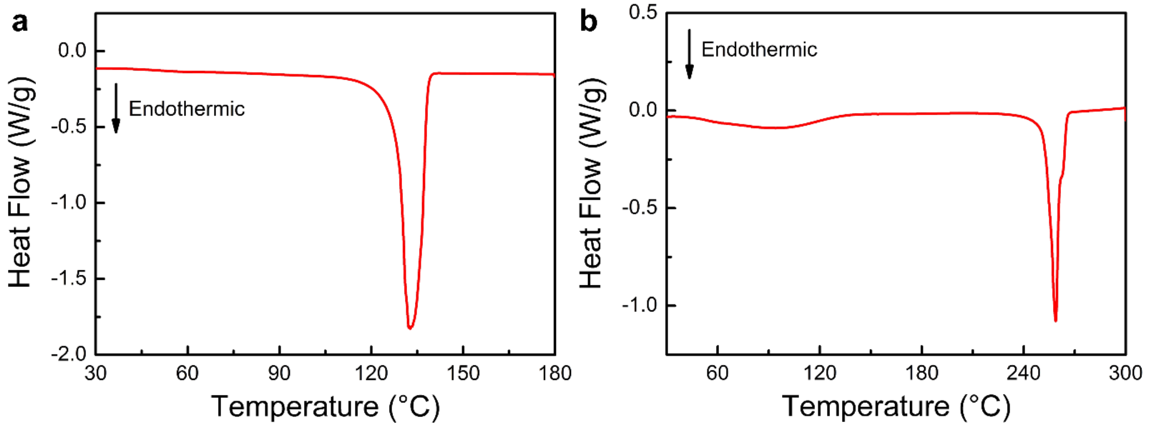

Figure S9. DSC curves of (a) polyethylene and (b) nylon 6, 6 fibres for a heating rate of 5 ℃ min^{-1} in N_2 atmosphere.

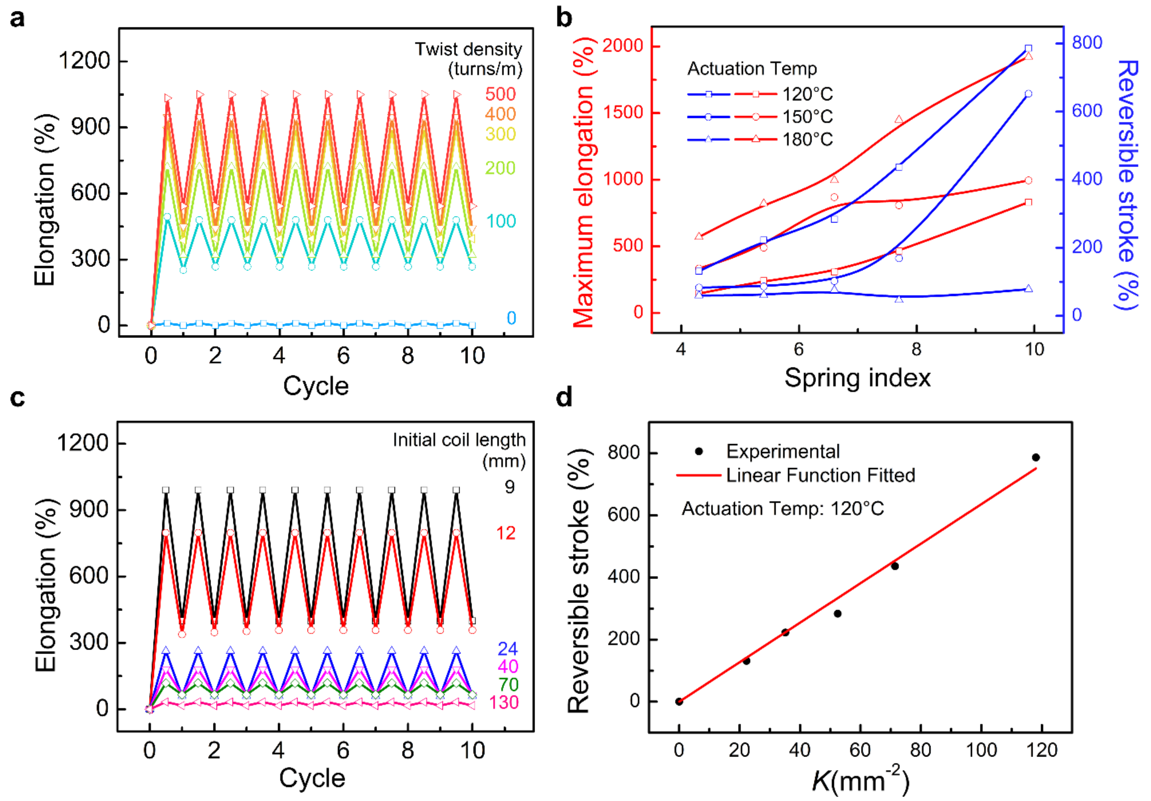

Figure S10. Actuator performance for coiled heterochiral nylon 6 fibre muscles as a function of (a) twist density, (b) spring index, and (c) initial coil length. (d) The calculated curve for the results in (b) for an actuation temperature of 120 ℃, assuming that stroke is proportional to the square of spring index. Fibres with diameter of 0.45 mm were annealed at 180 ℃ for one hour. For (a), the spring index was 6.6, and the actuation temperature was 150 ℃. For (b), the twist density was 5.42 turns cm^{-1}. For (c), the spring index was 6.6, the twist density was 5.42 turns cm^{-1}, and the actuation temperature was 180 ℃.

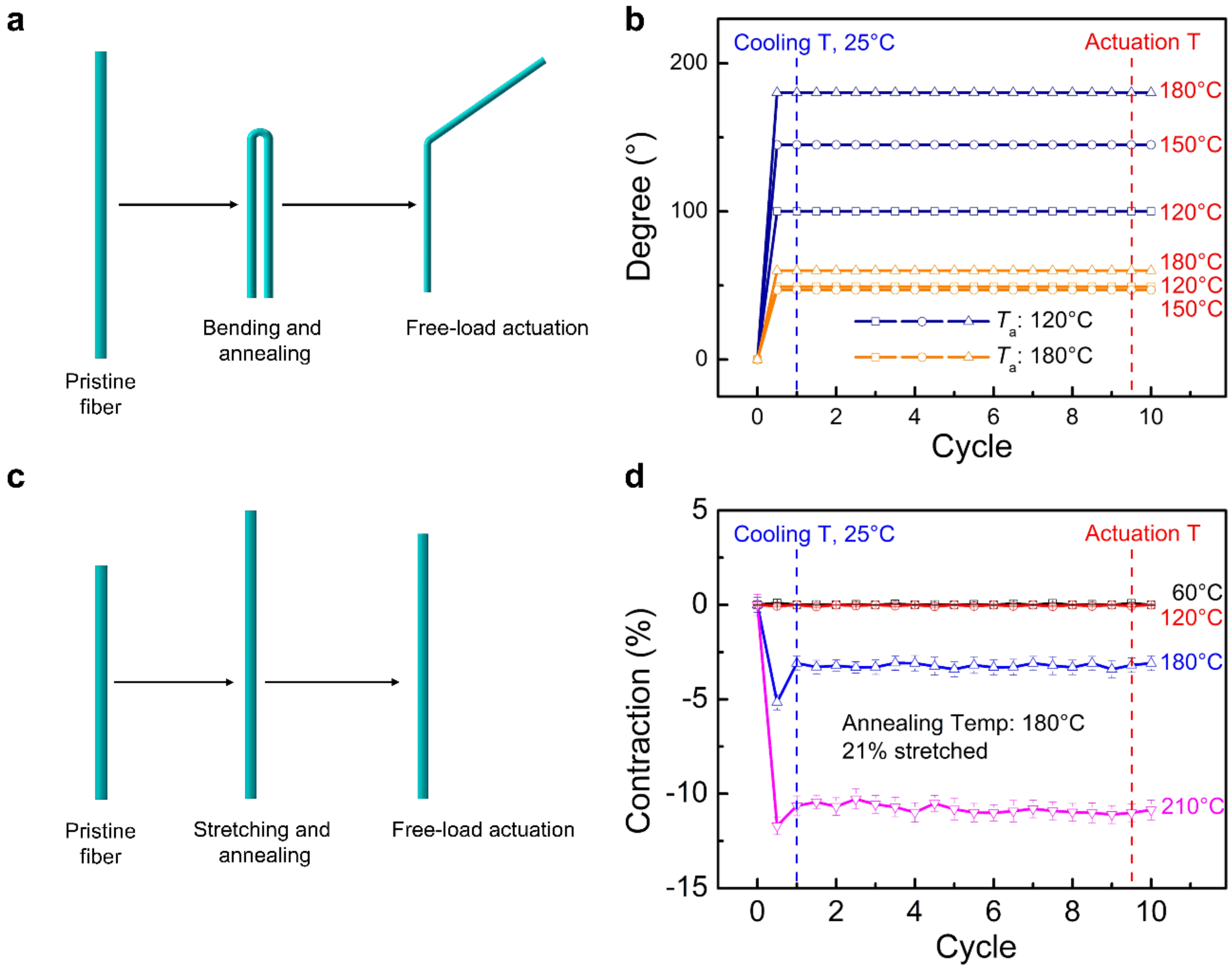

Figure S11. Fabrication and actuation for bent and stretched nylon 6 fibres. (a) Schematic illustration for thermal annealing of folded, non-twisted nylon 6 fibres. (b) Degree of bending during thermal actuation at different temperatures for folded nylon 6 fibres that have been thermally annealed at 120 and 180 ℃ for one hour. (c) Schematic illustration for thermal annealing of stretched, non-twisted nylon 6 fibres. (d) Contraction during heating/cooling cycles during actuation at different temperatures for 21% pre-stretched nylon 6 fibres that have been annealed at 180 ℃ for one hour. For comparison, we bent non-twisted nylon 6 fibres and annealed them at different temperatures (120 and 180 ℃) for one hour to fix the shape. They unbent irreversibly, and no reversible stroke was observed by heating the bent fibres, independent of the investigated actuation temperature (a, b). When a pristine fibre was stretched to 21% and thermally annealed at 180 ℃ to fix the shape, they showed nearly irreversible actuation at actuation temperatures of 180 and 210 ℃ (with very small fluctuations in the following cycles), and negligible length changes were observed during heating/cooling cycles at 60 and 120 ℃, indicating that twisting and coiling are necessary to obtain a large actuation stroke by magnifying the length change of the fibre muscle (c, d).

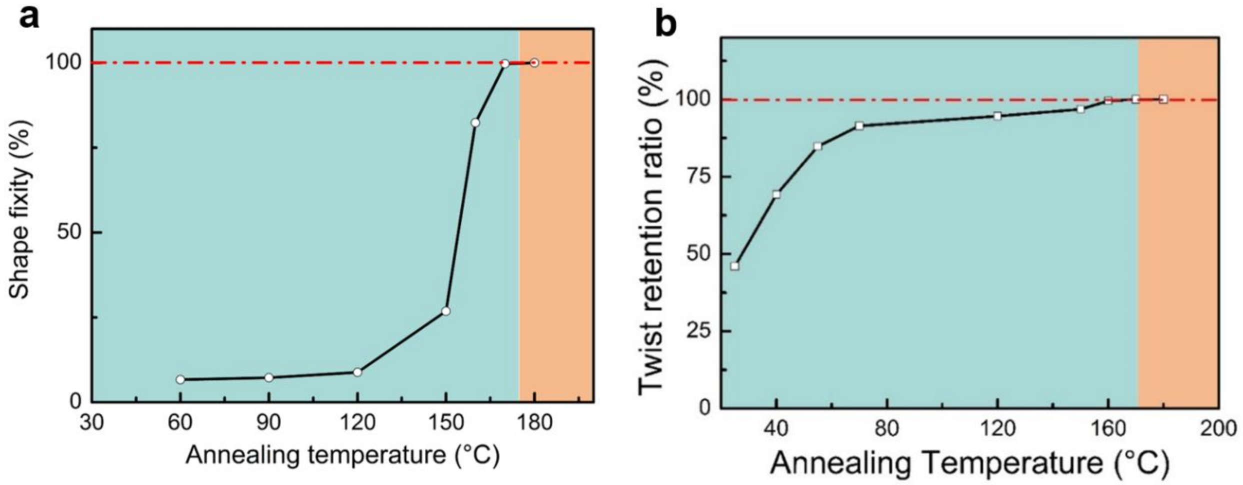

Figure S12. (a) Shape fixity for heterochiral coiled nylon 6 fibre muscles as a function of annealing temperature. The fibre diameter was 0.45 mm, and a fibre with twist density of 5.42 turns cm^{-1} was heterochirally coiled around a mandrel with diameter of 3 mm. The fibre muscle was thermally annealed for one hour at different temperatures with both ends tethered, and then was allowed to undergo length change at room temperature by removing tethering. The shape fixity is defined as the ratio of the tethered coil length obtained by mandrel coiling to the non-tethered coil length after annealing (and after 24 hours at room temperature) (b) Twist retention ratio as a function of annealing temperature for twisted, but non-coiled, nylon 6 fibre muscle. The fibre diameter was 0.45 mm, and the twist density was 5.42 turns cm^{-1}. The fibre muscle was thermally annealed for one hour at different temperatures with both ends tethered, and then was allowed to undergo twist release at room temperature by removing tethering. The twist retention ratio for the twisted, thermally annealed fibre muscle was calculated as the ratio of the retained twist to the initially inserted twist, where the retained twist was obtained by subtraction of released twist in 24 hours at room temperature from the initial inserted twist.

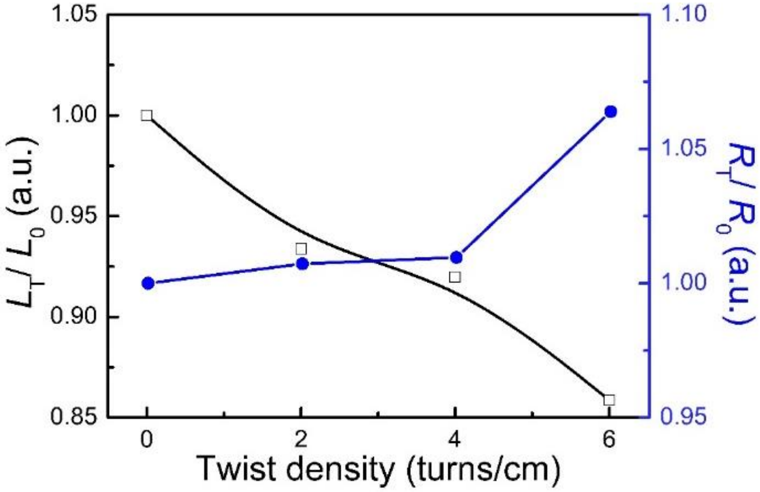

Figure S13. The ratio of length and radius of twisted, annealed nylon 6 fibre muscle to non-twisted, annealed nylon 6 fibre as a function of twist density. The non-tethered muscles were annealed at 180 ℃ for one hour.

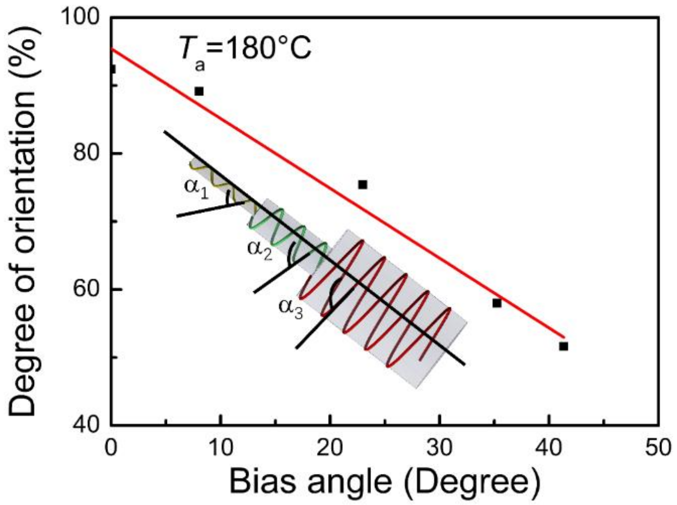

Figure S14. Degree of crystal orientation as a function of surface bias angle. The nylon 6 fibre muscles having different twist densities, were thermally annealed at 180 ℃ for one hour. Inset: schematic illustration showing that the bias angle decreases with decreasing radial diameter. The surface bias angle (α) was calculated from the twist density (T) and fibre diameter (d) using the following equation: $\alpha=\tan^{-1}(\pi dT)$.

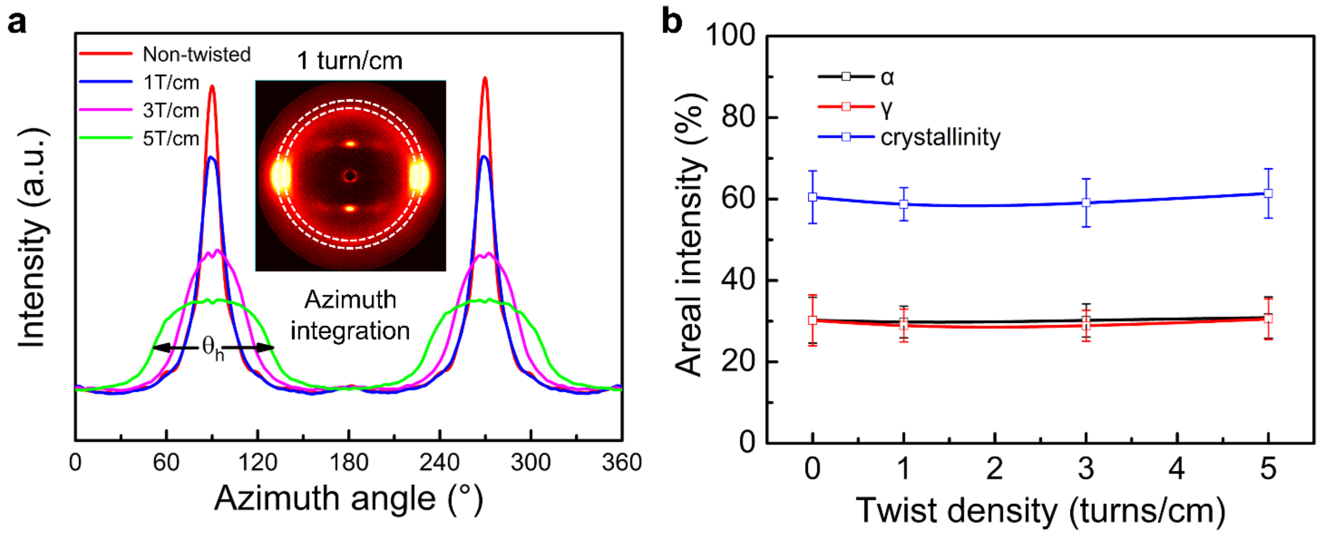

Figure S15. The azimuthal curves and crystallinity of nylon 6 fibres with varying twist density. (a) The azimuthal curves for nylon 6 fibres with varying twist density after annealing at 180 ℃. Inset: diffraction patterns in 2D WAXS for nylon 6 fibres annealed at 180 ℃ with 1 turn cm^{-1} inserted twists. The fibre direction is vertical. (b) The WAXS-based crystallinity of nylon 6 fibres with different inserted twist, following annealing at 180 ℃ for one hour.

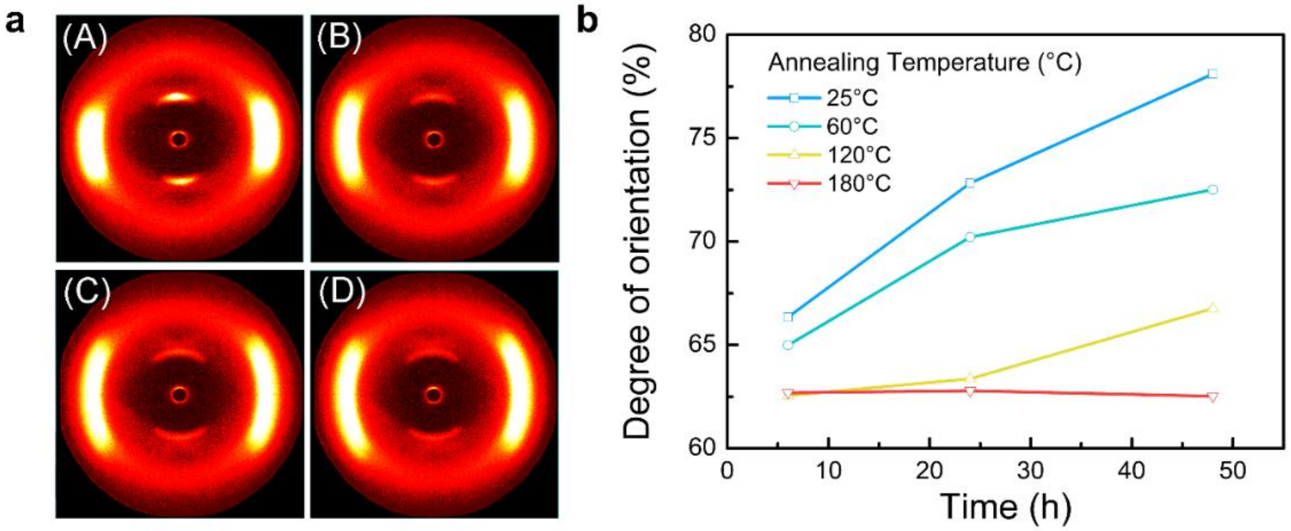

Figure S16. Degree of orientation as a function of annealing temperature and time. (a) The 2D WAXS patterns for 0.4 mm-diameter, twisted nylon 6 fibre muscles that have been annealed at 25 ℃ (A), 60 ℃ (B), 120 ℃ (C), and 180 ℃ (D) for one hour. The fibres were non-tethered at room temperature for 48 hours before testing. (b) The degree of orientation as a function of time for the twisted, annealed nylon 6 fibre muscles when they were non-tethered at room temperature. The initial inserted twist of the fibre muscles was 6 turns cm^{-1}.

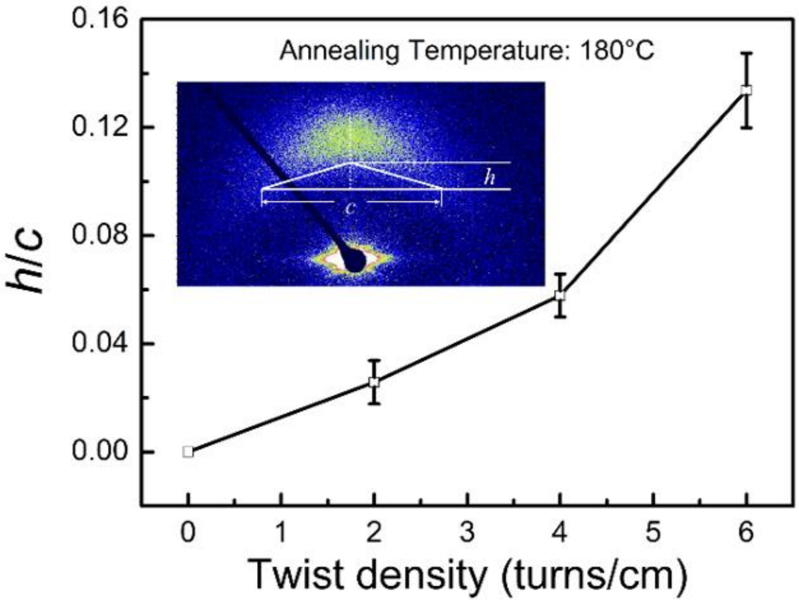

Figure S17. The sagitta-to-chord-length ratio (*h*/*c*) of the inner arc for twisted, annealed nylon 6 fibre muscles as a function of twist density. The inset shows the schematic illustration of calculation of *h*/*c* obtained from 2D SAXS scattering patterns. The 0.4-mm-diameter fibres in this figure were annealed at 180 ℃ for one hour while tethered.

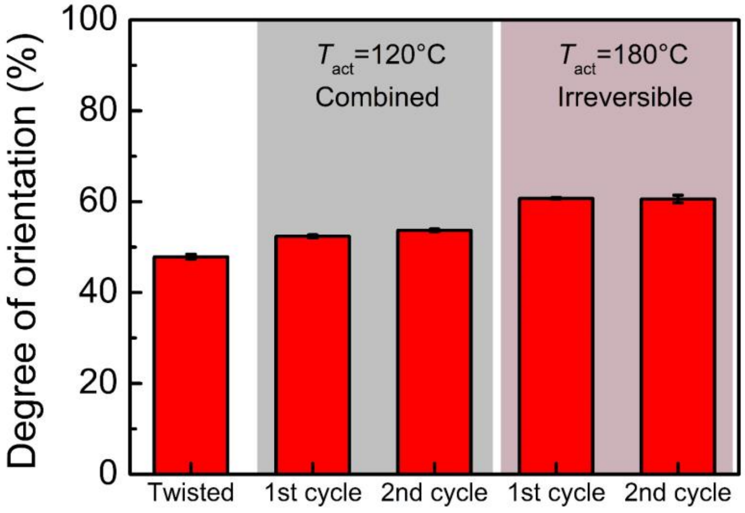

Figure S18. Degree of orientation of nylon 6 fibres after annealing at 150 ℃ and subsequent actuation when non-tethered. The degree of orientation for 0.2-mm-diameter twisted nylon 6 fibre muscles that were annealed at 150 ℃ for one hour before actuation, after actuation at 120 ℃ for one and two cycles, and after actuation at 180 ℃ for one and two cycles. The twist density of the nylon 6 fibre muscles was 14.0 turns cm^{-1} before actuation.

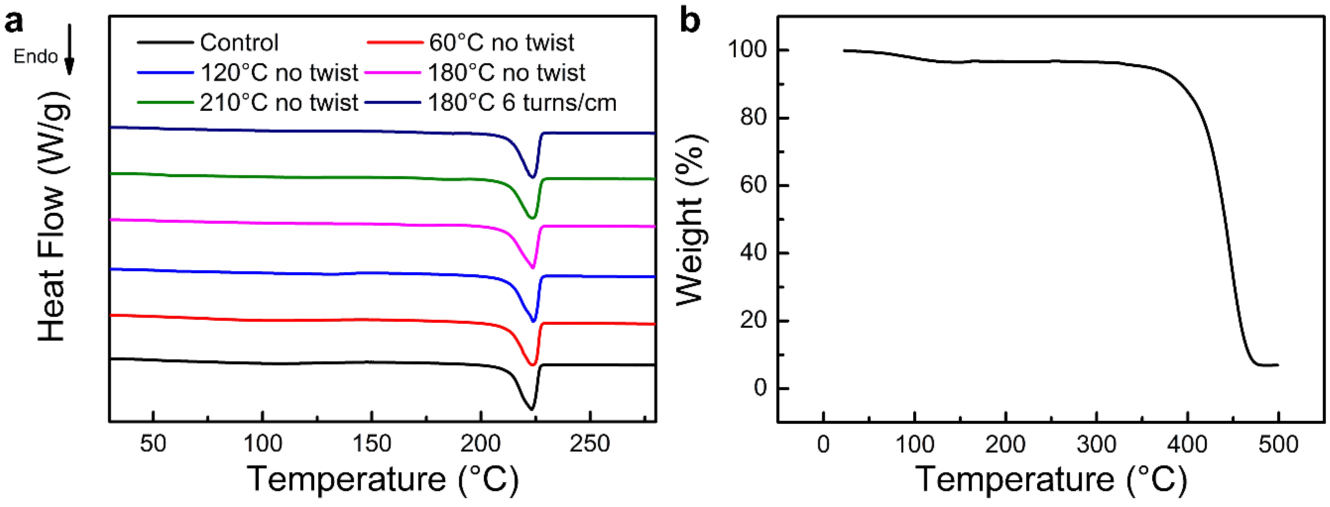

Figure S19. DSC and TGA curves of nylon 6 fibres. (a) DSC analysis at a temperature scan rate of 5 ℃ min^{-1} for non-twisted nylon 6 fibres that were thermally annealed at 60, 120, 180, and 210 ℃ for one hour, and a twisted nylon 6 fibre muscle (6.0 turns cm^{-1}) that was thermally annealed at 180 ℃ for one hour, using a non-twisted fibre as a control. (b) Thermogravimetric analysis curve for the non-twisted nylon 6 fibre.

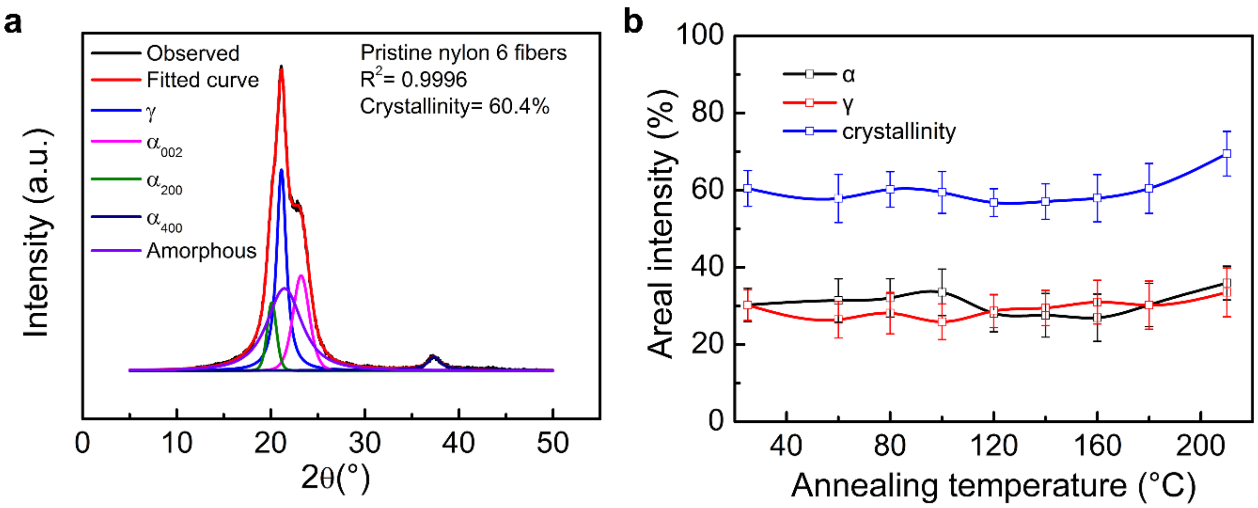

Figure S20. Crystallinity of nylon 6 fibres calculated by WAXS. (a) The intensity of diffraction peaks versus Bragg angle (2θ) in one dimensional WAXS for nylon 6 fibres, and the peak fitting for amorphous and crystalline peaks (α and γ peaks). (b) The contents of the crystalline α phase and γ phase for non-twisted nylon 6 fibres that have been annealed for one hour at different temperatures. The error bars correspond to the standard deviation for measurements on three different samples. The measurements were conducted at room temperature for this and the following X-ray figures.

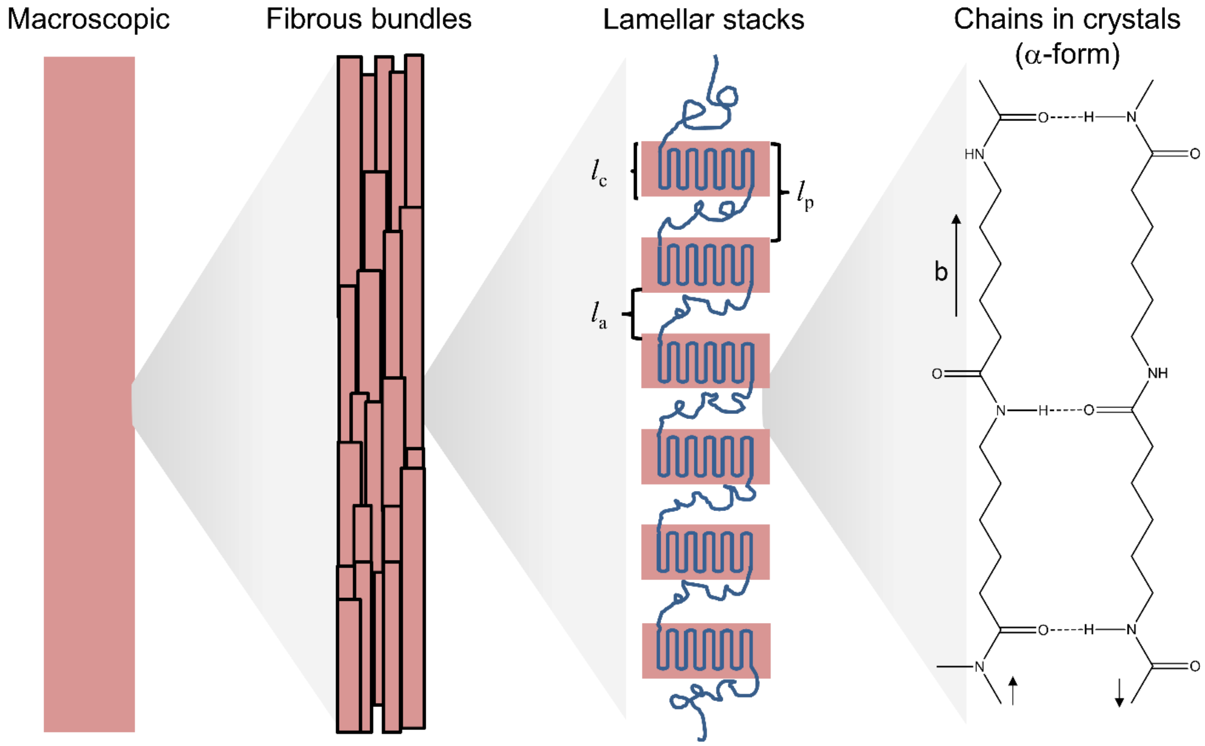

Figure S21. Schematic illustration, for different length scales, of the structure of a nylon 6 fibre. From left to right, are the nylon fibre on the millimeter scale, fibrous bundles on the micrometer scale, the periodic crystalline regions and amorphous regions on the nanometer scale, and the polymer chain structure.

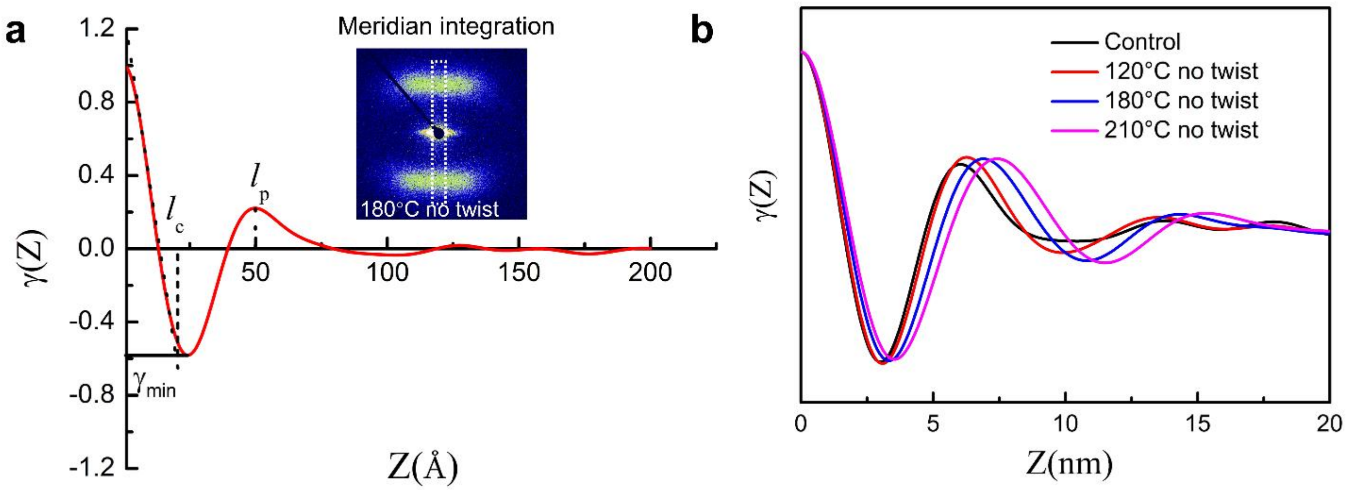

Figure S22. The 1D correlation curve of nylon 6 fibres. (a) A modeling 1D correlation curve obtained from meridian integration and inverse Fourier transformation of 2D SAXS signals, from which l_p, l_c, and l_a can be obtained. Inset: the rectangular area for meridian integration. (b) The 1D correlation curves derived from 2D SAXS for nylon 6 fibres with different annealing temperatures without inserting twists. The thermal annealing time was one hour.

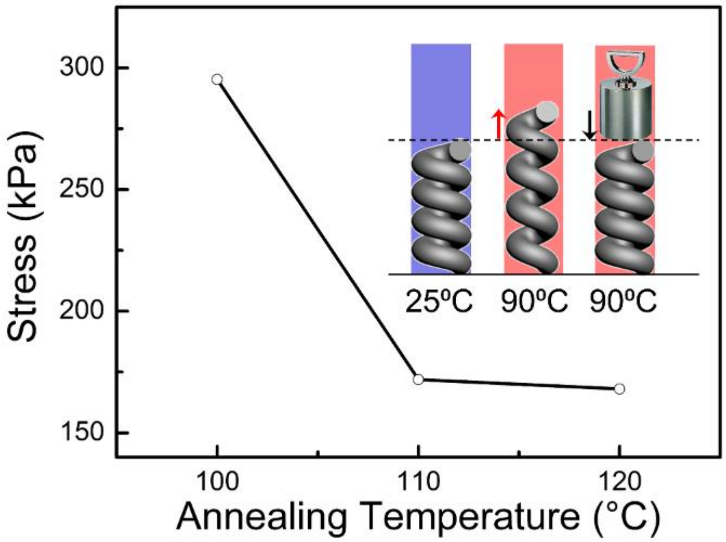

Figure S23. The compressive stress as a function of annealing temperature for coiled heterochiral polyethylene fibre muscles. The compressive stress used to keep the length unchanged as a function of annealing temperature for heterochiral coiled, annealed polyethylene fibre muscles, when these muscles were actuated at 90 ℃. The inserted twist was 3.75 turns cm^{-1}, the spring index was 6.0, and the annealing time was one hour. The measured T_s of polyethylene was ~110 ℃.

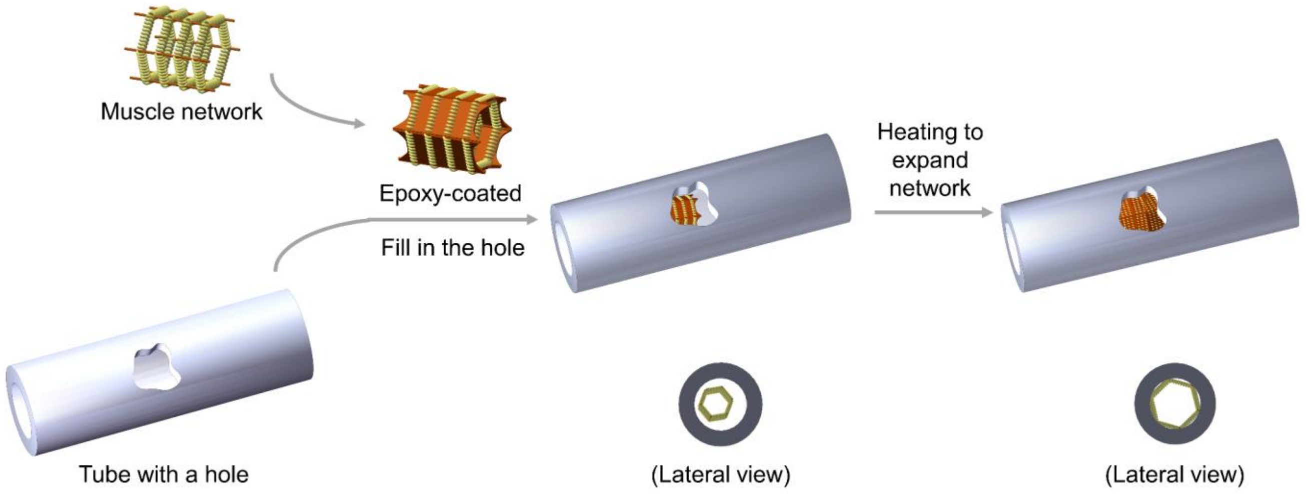

Figure S24. Schematic illustrations of expandable network formed by heterochiral coiled muscles with an irreversible actuation mode, and their use to fix a hole in a leaking tube.

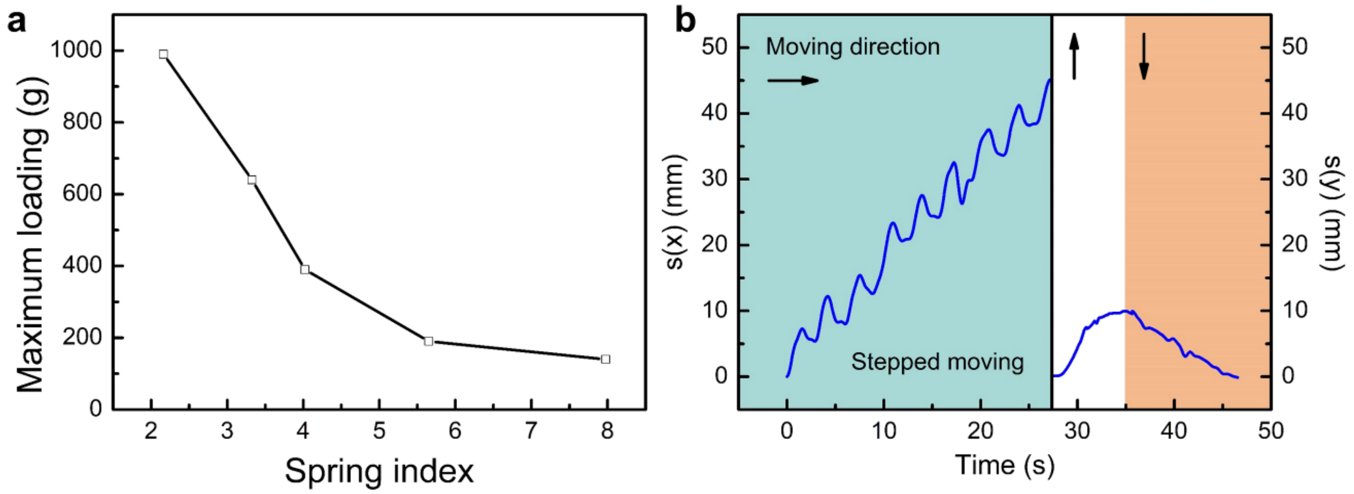

Figure S25. Quantitative analysis of electrically-driven grippers and biomimetic worms. (a) The maximum loading of the electrically driven gripper shown in Figure 4 as a function of spring index (Movie S9). The fibre diameter and the gripper diameter of the nylon 6 muscle are 0.43 mm and 2.0 cm, respectively. (b) The translation distance as a function of time, for forward (left panel) and bi-directional displacement left and right (right panel) for electrically-driven biomimetic worms in Movie S10. The arrows pointing right, up, and down means the biomimetic worm moving forward, left, and right, respectively.

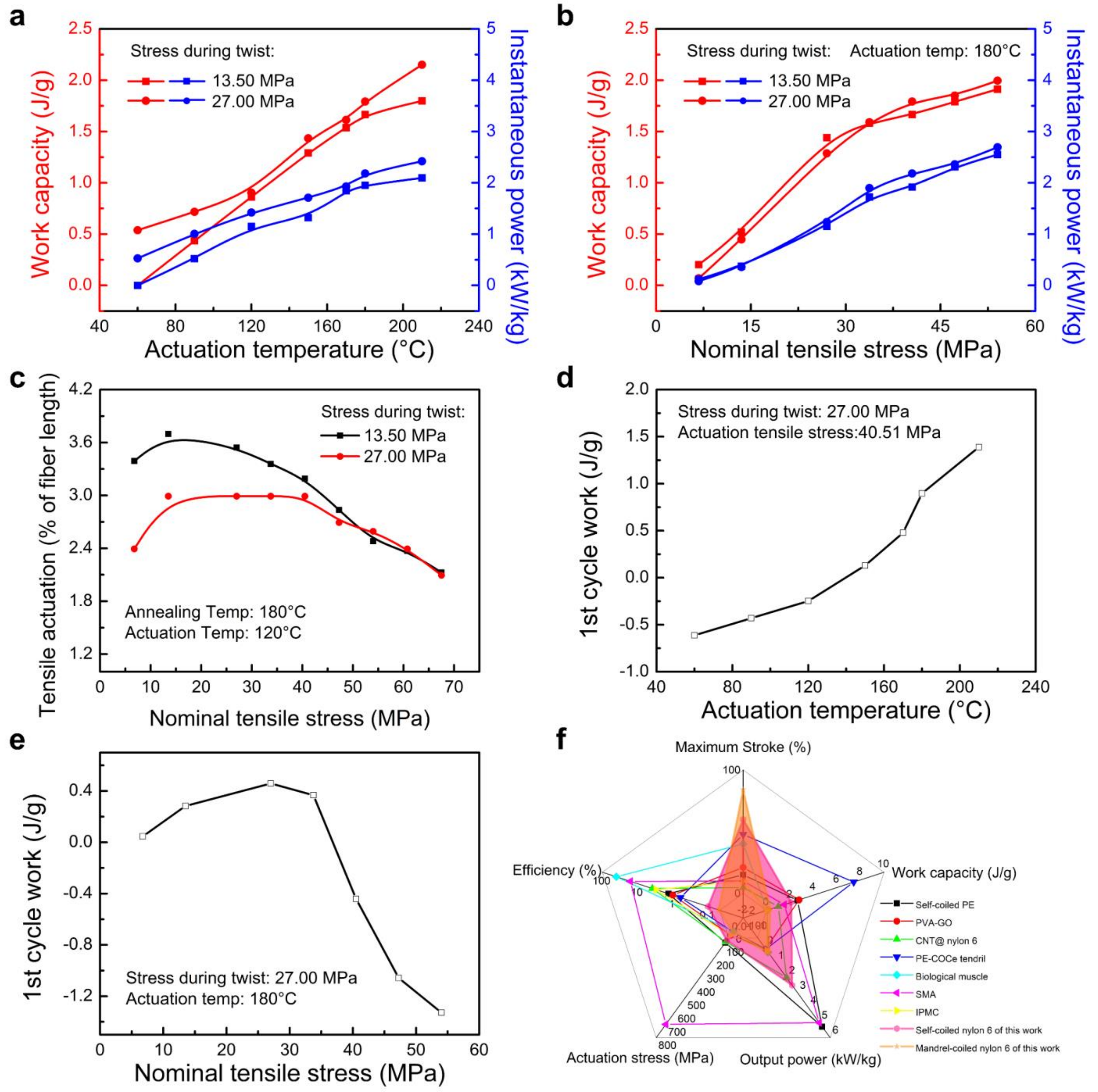

Figure S26. Actuator performance for reversible and first cycle actuation for self-coiled nylon 6 muscles. (a-c) The mechanical work capacity and instantaneous power as a function of (a) actuation temperature and (b) tensile load, and the tensile stroke as a function of (c) tensile load for the reversible cycles. (d, e) The contractile work during the first actuation cycle as a function of (d) actuation temperature and (e) tensile load. For (a) to (e), the nylon 6 muscles were annealed at 180 ℃ for one hour. The heating rate was about 75 ℃ min^{-1}. For (b) and (e), the actuation temperature was 180 ℃. (f) Comparison of reversible work capacity, actuation stress, output instantaneous power, efficiency, and maximum stroke of the nylon 6 muscles in this work with other materials in the literature.

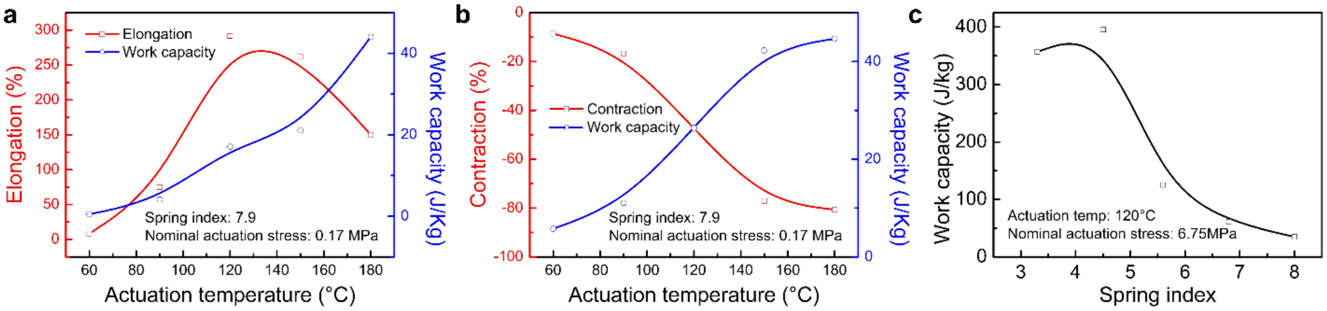

Figure S27. Actuation strain and contractile work capacity for mandrel-coiled nylon 6 fibres. (a, b) Tensile strokes and work capacity for (a) heterochiral and (b) homochiral fibres as a function of actuation temperature for a spring index of 7.9 and a low actuation stress of 0.17 MPa. (c) Work capacity of homochiral nylon 6 fibres as a function of spring index for an actuation temperature of 120 ℃ and an actuation stress of 6.75 MPa. For (a) to (c), a 0.45 mm-diameter fibre was twisted to provide a density of 5.42 turns cm^{-1}, mandrel coiled, and annealed at 180 ℃ for one hour.

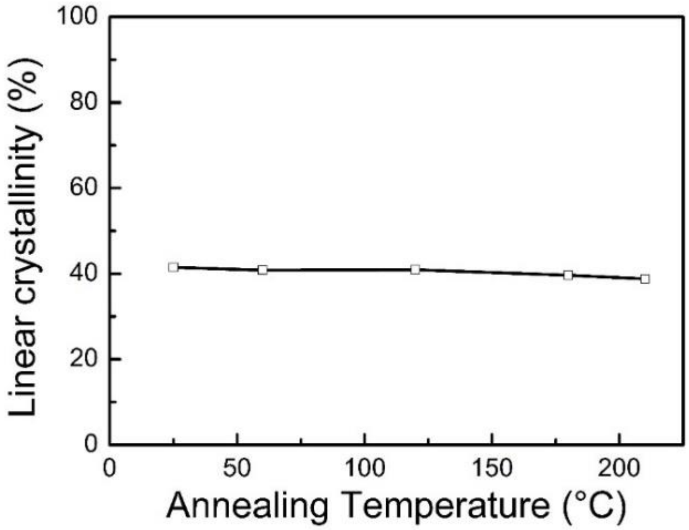

Figure S28. Linear fraction of crystalline regions (ϕ_c) of non-twisted 0.4-mm-diameter-nylon 6 fibres that have been annealed at different temperatures for one hour, which was calculated from $\phi_c = l_c/l_p$ using the data in Figure 3b in main text.

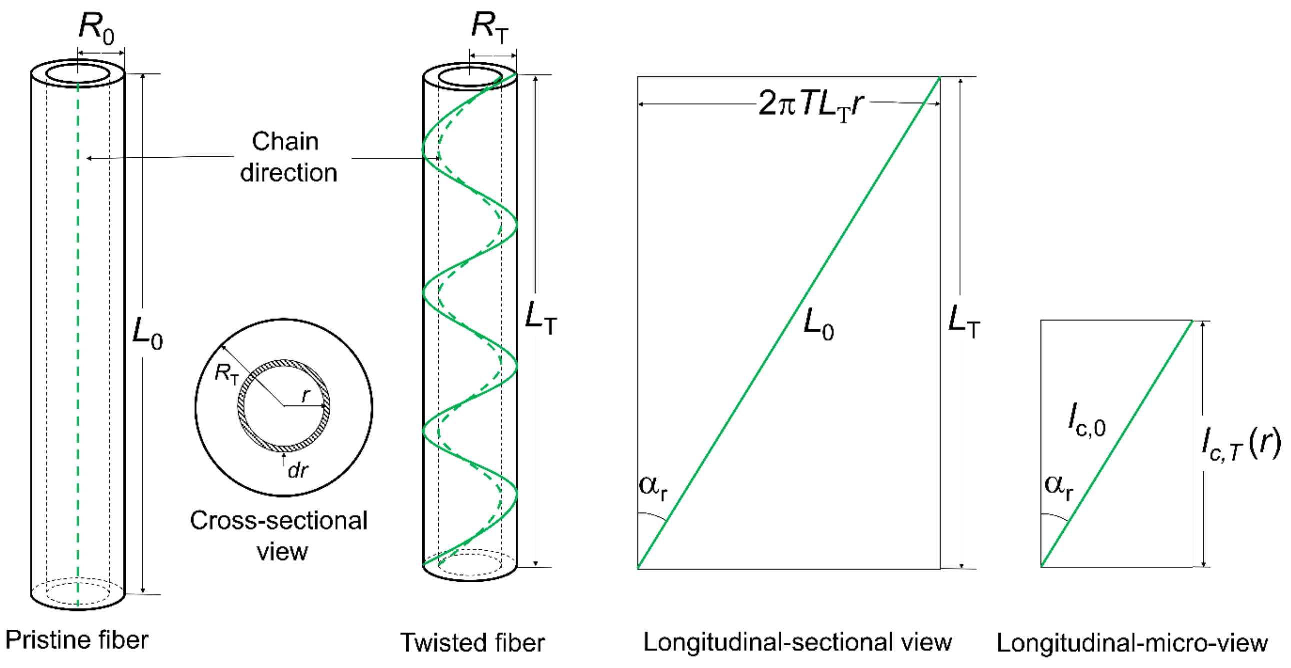

Figure S29. Schematic illustration for calculation of the periodic lengths of lamellar crystals ($l_{c,T}$) at an inserted twist (T) from that of the non-twisted fibre ($l_{c,0}$).

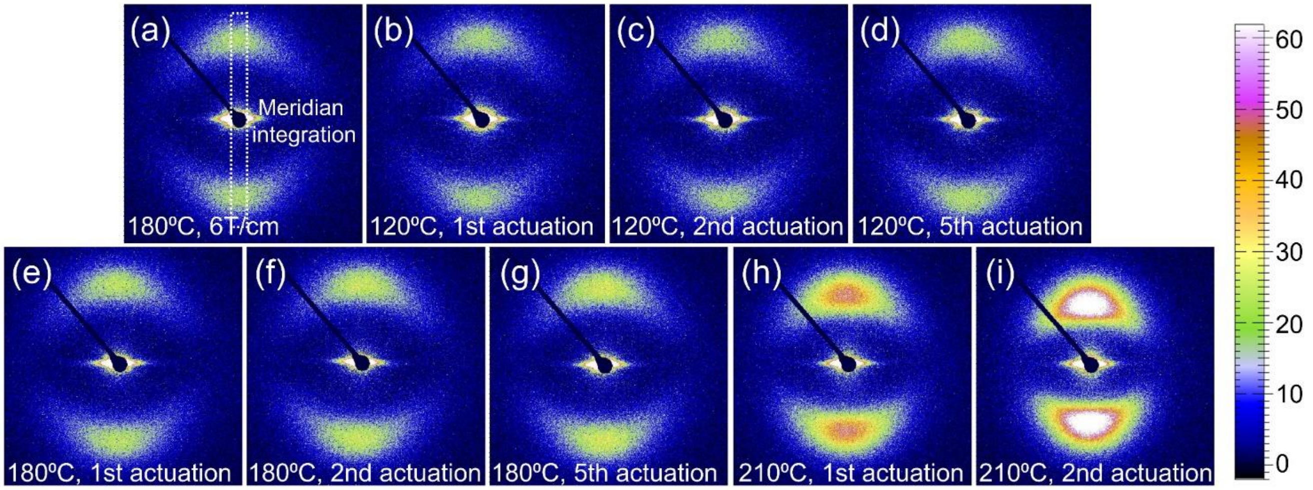

Figure S30. The 2D SAXS patterns for twisted, annealed nylon 6 fibre muscles before actuation (a), after actuation at 120 ℃ for (b) 1st, (c) 2nd, and (d) 5th cycles, after actuation at 180 ℃ for (e) 1st, (f) 2nd, and (g) 5th cycles, and after actuation at 210 ℃ for 1st (h) and (i) 2nd cycles. The fibres in this figure were annealed at 180 ℃ for one hour while tethered. The fibre direction was vertical.

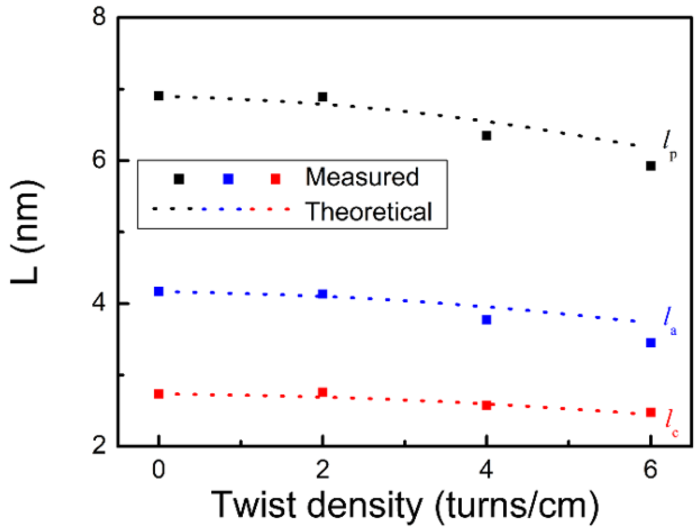

Figure S31. Twist-induced changes in the long period length and arc patterns obtained by SAXS. Theoretically calculated and experimentally measured values of l_c, l_a, and l_p for twisted, annealed nylon 6 fibre muscles as a function of twist density. The 0.4-mm-diameter fibres in this figure were annealed at 180 ℃ for one hour while tethered.

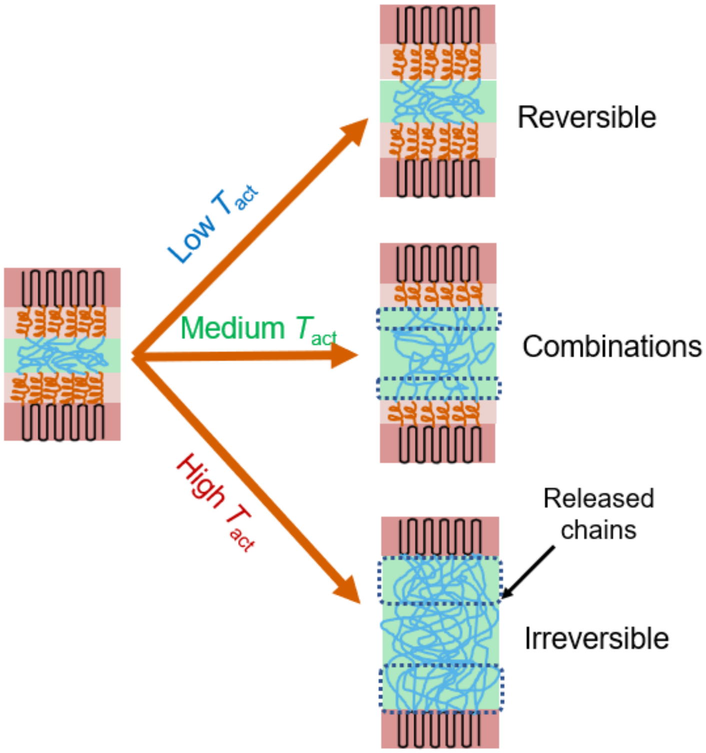

Figure S32. A schematic to demonstrate the process of the orientation change of the chain segments.

Supplementary Table S1. Comparison of performance metrics of some typical artificial muscles.[a]

Materials	Stimulus	Maximum Stroke	Work capacity ($J\ g^{-1}$)	Output power ($kW\ kg^{-1}$)	Actuation speed	Actuation stress	Efficiency (%)	Ref.
Self-coiled PE	Heat (60-120 ℃)	15% contraction	2.63	5.26	N/A	>85 MPa	1.32%	[1]
PVA-GO fibre	Heat (60-210 ℃)	21 $N{\cdot}m\ kg^{-1}$	2.76	0.069	600 rpm	N/A	1.0-1.5%	[9]
CNT- nylon 6 SRAM	Electrochemical (3 V, 1 Hz)	4.7% contraction	0.99	1.98	10% s^{-1}	80 MPa	3.8%	[10]
PE-COCe tendril	Heat	47.7% contraction	7.42	0.075-0.09	6.33 N s^{-1}	5 MPa	0.6%	[11]
Biological muscle	Biological signal	>40%	0.04	0.28	N/A	0.35-1 MPa	40%	[12]
SMA	Heat	10%	<1.5	<5	N/A	<700 MPa	<16%	[12]
IPMC	Voltage (usually <10 V)	<10%	0.004	0.2	~10%/s	30 MPa	<3%	[12]
DEA	High voltage	~400% (areal strain)	3.5 J cm^{-3}	N/A	N/A	<7.2 MPa	<90%	[12]
Self-coiled nylon 6	Heat (60-210 ℃)	60% contraction	2.1	2.5	3% s^{-1}	>70 MPa	<0.1%	This work
Mandrel-coiled nylon 6	Heat (60-210 ℃)	84% contraction	0.3	N/A	5% s^{-1}	6.75 MPa (C=7.9)	~0.05%	This work

[a] PE: polyethylene; SRAM: sheath-run artificial muscles; COCe: cyclic olefin copolymer elastomer; SMA: shape memory alloys; IPMC: ionic-polymer/metal composites; DEA: dielectric-elastomer actuators.

Supplementary Table S2. Melting enthalpy and crystallinity obtained by DSC analysis.

Samples	Annealing temperature (℃)	Melting temperature (℃)	Melting enthalpy ($J\ g^{-1}$)	Crystallinity (%)
Non-twisted nylon 6 fibre	non-annealed	223.0	75.5	39.7
	60	223.7	76.7	40.4
	120	223.9	75.9	40.0
	180	223.8	73.0	38.4
	210	223.5	79.8	42.0
Twisted nylon 6 fibre (6 turns cm^{-1})	180	223.7	74.5	39.2
Polyethylene fibre	non-annealed	133.0	166.0	57.8
Nylon 6, 6 fibre	non-annealed	259.3 (γ phase)<br>262.7 (α phase)	88.9	47.3

Movie S1. Artificial flower.

Description: Coiled heterochiral nylon 6 muscles dyed with red color can be assembled to form an artificial flower that can mimic flower blooming upon heating. The preparation of the coiled heterochiral muscles was as follows. A twisted 0.45-mm-diameter nylon 6 fibre was heterochirally wrapped around a mandrel and thermally annealed at 150 ℃ for one hour to set the shape. The twist density was 5.42 turns cm^{-1}, and the spring index was 6.6. Heating the assembly of the coiled nylon 6 heterochiral muscles to above 150 ℃ resulted in irreversible expansion of each coil, mimicking flower blooming.

Movie S2. Heterochiral double-coil muscle for fixing pipe leakage.

Description: The irreversible actuation mode of a heterochiral nylon 6 double-coil muscle can be used for fixing pipe leakage. The preparation of the double-coil muscle was as follows. A twisted 0.45-mm-diameter nylon 6 fibre muscle was heterochirally wrapped around a mandrel and thermally annealed at 150 ℃ for one hour to set the shape. The twist density was 5.42 turns cm^{-1}, and the spring index was 6.6. After thermal annealing, the coiled heterochiral nylon 6 muscle was further wrapped around a second mandrel to form a double-coil muscle. Six 0.8-mm-diameter parallel steel rods were attached on the surface of the double coil muscle in the length direction, and cotton yarns were used to tie the steel rods on the nylon 6 double-coil to set the shape. Heating the double-coil muscle to above 180 ℃ resulted in irreversible thermal expansion of the double-coil muscle in the radial direction. Such a double-coil muscle can be used to fix a leaking pipe. First, the double-coil muscle, carrying a liquid mixture of epoxy resin and its hardener on its surface, was inserted into the position of pipe leakage. Second, heating the double-coil muscle to above 180 ℃ expanded the double-coil muscle to contact the pipe surface, and simultaneously cure the epoxy resin to fix pipe leakage.

Movie S3. Inflatable polymer stent.

Description: A heterochiral nylon 6 double-coil muscle, with an irreversible actuation mode, was used to inflate a soft tube. A nylon 6 double-coil muscle, which was prepared according to the protocol in Movie S2, was used to inflate a soft tube upon heating to above 180 ℃. This demonstration shows the possibility of using such a heterochiral double coil muscle as an inflatable polymer stent for a vessel.

Movie S4. Automatic fire extinction tubes.

Description: A homochiral nylon 6 double-coil muscle with irreversible actuation mode was used for fire extinction. The homochiral nylon 6 double-coil muscle, which was prepared using a protocol similar to that in Movie S2, with the same chirality of fibre twisting and fibre coiling. Upon heating to above 180 ℃, the homochiral double-coil muscle contracted in the radial direction. Such a double-coil muscle wrapping on a fire-resistance film contracted upon heating by the fire, and extinguished the fire by removing or limiting its oxygen supply.

Movie S5. Liquid release at high temperature.

Description: A coiled homochiral nylon 6 muscle was used for releasing the liquid encapsulated by a fragile thin film. The coiled homochiral nylon 6 muscle was prepared using a protocol similar to that in Movie S1, with the same chirality of fibre twisting and fibre coiling. A thin styrene ethylene butylene

styrene (SEBS) film was spray coated on the surface of the homochiral coil muscle to make a capsule. Then dark-blue-colored water was injected into the capsule using a syringe and sealed by wax. Dropping the liquid-containing coiled muscle into a water bath at 80 ℃ contracted the homochiral muscle and broke the encapsulated SEBS sheath, resulting in release of the encapsulated liquid. This demonstration showed the possibility of using such a homochiral coil for controllable liquid release.

Movie S6. Electrically driven opening and closing of umbrella.

Description: A heterochiral nylon 6 coil muscle with reversible actuation mode was used for electrically controlled reversible opening of an umbrella. The nylon 6 fibre and a 0.05-mm-diameter copper wire were plied together, heterochirally wrapped around a mandrel, and thermally annealed at 180 ℃ for one hour to set the shape. The twist density was 5.42 turns cm^{-1}, and the spring index was 6.6. A 0.33 V cm^{-1} square wave potential (normalized to the wire length) was applied to the wire to open or close the umbrella.

Movie S7. Electrically driven ventilation system.

Description: A coiled heterochiral nylon 6 muscle with reversible actuation mode was used for electrically controlled reversible opening of roof to control ventilation. The coiled heterochiral nylon 6 muscle was prepared according to the protocol in Movie S6, and a 0.33 V cm^{-1} square wave potential (normalized to wire length) was applied to the wire to open or close the roof to control the ventilation.

Movie S8. Crawling soft robot.

Description: A reversibly actuating, coiled heterochiral nylon 6 muscle was used for an electrically controlled crawling soft robot. The coiled heterochiral nylon 6 muscle was prepared according to the protocol used for Movie S6, and inserted with a 4-mm-diameter wood rod with a zig-zag surface pattern. The coiled muscle can move forward upon heating/cooling by applying/removing a 0.33 V cm^{-1} square wave potential (normalized to wire length).

Movie S9. Electrically controlled grippers.

Description: A coiled heterochiral nylon 6 muscle with reversible actuation was used for electrically controlled grippers. The coiled heterochiral nylon 6 muscle was prepared using the protocol used for Movie S6. The coiled heterochiral muscle was connected end-to-end to form a ring. Applying a 0.33 V cm^{-1} square wave potential (normalized to wire length) opened the gripper to enable it to wrap around the bottle's neck. Removing the potential closed the gripper, which enabled the bottle to be lifted.

Movie S10. Moving-direction-controlled soft robot.

Description: Two individually controlled, parallel-aligned coiled heterochiral nylon 6 muscles with reversible actuation were used for preparing an electrically-controlled crawling soft robot. Two heterochiral coiled nylon 6 muscles were prepared using the same protocol as used for Movie S6, and they were assembled in parallel to make a crawling robot. Each coil muscle can be individually controlled by electrical heating. The soft crawling robot can crawl forward on a surface with zig-zag pattern by applying/removing a 0.33 V cm^{-1} square wave potential (normalized to wire length). Applying potential to one of the two wire coils can change the movement direction.

Movie S11. A grow-able crawling soft robot that can change its initial length.

Description: A PE fiber and 0.03-mm copper wire were twisted and plied together, heterochirally wrapped on around a mandrel, and thermally annealed at 100 ℃ for 1 h to set the shape. The twist density was 5.42 turns cm^{-1}, and the spring index was 9.5. Then it was used to prepare a soft crawling robot, by assembling two feet on the coil. The heterochiral muscle with conductive path can walk forward on a wedge-shape road by applying/removing a 0.1 V cm^{-1} square wave potential (normalized to wire length) to heat the muscle to 50 ℃. The muscle can grow to another muscle with an elongated length by applying a 0.3 V cm^{-1} potential to heat the muscle to 80 ℃ so that it can pass a trench with 20 mm in width and 3 mm in depth.

Supplementary References

1. Haines C S, Lima M D, Li N, *et al*. Artificial muscles from fishing line and sewing thread. *Science*, 2014; **343**(6173): 868-872.
2. Mirvakili S M, Hunter I W. Fast torsional artificial muscles from NiTi twisted yarns. *ACS Appl Mater Inter*, 2017; **9**(19): 16321-16326.
3. Xu J R, Ren X K, Yang T, *et al*. Revisiting the thermal transition of β-form Polyamide-6: evolution of structure and morphology in uniaxially stretched films. *Macromolecules*, 2018; **51**(1): 137-150.
4. Strobl G R, Schneider M. Direct evaluation of the electron density correlation function of partially crystalline polymers. J Polym Sci Pol Phys. 1980; 18(6): 1343-1359.
5. Jonas A M, Russell T P, Yoon D Y. Synchrotron x-ray scattering studies of crystallization of poly (ether-ether-ketone) from the glass and structural changes during subsequent heating-cooling processes. *Macromolecules*, 1995; **28**(25): 8491-8503.
6. Foroughi J, Spinks G M, Wallace G G, *et al*. Torsional carbon nanotube artificial muscles. *Science*, 2011; **334**(6055): 494-497.
7. Rafique F Z, Vasanthan N. Crystallization, crystal structure, and isothermal melt crystallization kinetics of novel polyamide 6/SiO2 nanocomposites prepared using the sol–gel technique. *J Phys Chem B*, 2014; **118**(31): 9486-9495.
8. Murthy N S, Grubb D T. Deformation of lamellar structures: simultaneous small ‐ and wide ‐ angle x ‐ ray scattering studies of polyamide ‐ 6. J Polym Sci Pol Phys. 2002,;**40**(8): 691-705.
9. Yuan J, Neri W, Zakri C, *et al*. Shape memory nanocomposite fibers for untethered high-energy microengines. *Science*, 2019; **365**(6449): 155-158.

10. Mu J, Jung de Andrade M, Fang S, *et al*. Sheath-run artificial muscles. *Science*, 2019; **365**(6449): 150-155.
11. Kanik M, Orguc S, Varnavides G, *et al*. Strain-programmable fiber-based artificial muscle. *Science*, 2019; **365**(6449): 145-150.
12. Mirvakili S M, Hunter I W. Artificial muscles: Mechanisms, applications, and challenges. *Adv Mater*, 2018; **30**(6): 1704407.
